# Transposable Element–Driven *PIEZO* Mutation Enhances Locust Flight in Plateau Hypoxia

**DOI:** 10.1002/advs.76705

**Published:** 2026-07-20

**Authors:** Xuanzhao Li, Ying Liu, Longsheng Xing, Xianliang Huang, Yingming Sun, Lei Yue, Yuze Zhang, Huilong Du, Bing Chen

**Affiliations:** ^1^ College of Life Sciences Hebei University Baoding China; ^2^ Hebei Basic Science Center for Biotic Interaction Hebei University Baoding China

**Keywords:** migratory locust, PIEZO, Qinghai‐Tibet Plateau, selective sweep, transposable element

## Abstract

Transposable elements contribute to species diversification and adaptive evolution. However, it remains challenging to reconcile the impacts of TEs on microevolution (within species) and macroevolution (among species) given their huge pool and complex dynamics in genomes. Here, we generated the genome for the notorious worldwide migratory locust, *Locusta migratoria*, sampled from Qinghai‐Tibet Plateau. Comparative genome analyses uncovered extensive TE‐driven genome size variation, and lineage‐specific expansion of TEs, particularly of the PiggyBac superfamily in the locust. Whole‐genome resequencing of 153 locust individuals collected across an altitudinal gradient ranging from 2 to 4100 m clarified their distinct elevational genetic divergence, which is consistent across SNP and TE insertion polymorphisms analysis. A total of 11 869 TE insertions were found within selective sweeps, including at least 279 genes associated with altitude adaptation. TEs from five superfamilies, accounting for 55.68% of the candidate adaptive TEs, exhibited significant expansion in both microevolution and macroevolution. We validated that PiggyBac insertion into the *PIEZO* gene induced alternative splicing and enhanced flight performance under hypoxia through AMPK pathway. These data provide robust evidence that TEs are central drivers of genomic evolution across micro‐ and macroevolutionary scales, shaping genomic landscape and promoting adaptive change.

## Graphical abstract

Why transposable elements (TEs) persisted or expanded in genomes remains a mystery. Using integrated analysis of TE macro‐ and microevolution in locusts, our results showed that thousands of TE insertions promoted widespread adaptive variation. Subfamilies of candidate adaptive TEs amplified and reshaped species‐level genomic architecture. Functional study revealed TE‐mediated mutations in mechosensitive channel gene *PIEZO*, enhancing locust flight performance under hypoxia.

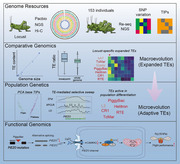

## 1 Introduction

Transposable elements (TEs) account for a major fraction of eukaryotic genomes and represent a powerful force shaping genome evolution. Their insertions can generate structural variation, influence gene expression, and alter epigenetic states, ultimately exerting profound impacts on phenotypic diversity [1, 2, 3]. Notably, TE insertion polymorphisms (TIPs) display genome‐wide population dynamics. TIPs constitute an important source of genetic variation and likely contribute to epigenomic and transcriptional differences, thus providing a potential substrate for local environmental adaptation [1, 4, 5]. Despite their pervasive influence, it remains challenging to reconcile the roles of TEs across microevolutionary processes within species and macroevolutionary patterns among species, owing to their vast diversity and complex dynamics.

The migratory locust, *Locusta migratoria*, provides an exceptional model for investigating the contribution of TE‐derived variation to microevolutionary processes associated with high‐altitude adaptation. TEs constitute approximately 65% of the locust genome (∼6.3 Gb), which is one of the largest sequenced arthropod genomes [6, 7, 8]. TIPs have been documented among geographically distinct locust populations, indicating potential TE‐derived genetic variation [9]. In China, the migratory locust occupies a broad elevational range, spanning low‐altitude plains to the Qinghai‐Tibet Plateau (QTP) with average altitude exceeding 4000 m [10]. Locust populations have colonized the QTP for more than 34 000 years and exhibit morphological, physiological and genetic adaptation to the extreme plateau environments [11, 12]. However, the mechanisms by which TE‐derived genetic variation contributes to high‐altitude adaptation in locusts remain poorly understood.

High‐altitude adaption in animals involves physiological and metabolic changes in response to shortage of ambient oxygen supply and air pressure [11]. The mechanosensitive channel gene *PIEZO* could be a strong candidate regulating oxygen responses, especially during flight. *PIEZO* proteins have been documented as components of excitatory ion channels directly gated by mechanical forces [13, 14]. In vertebrates, the *PIEZO* family comprises two paralogs, *PIEZO*1 and *PIEZO*2, whereas most invertebrate genomes harbor a single *PIEZO* homolog. *PIEZO* channels are activated by a wide range of mechanical stimuli, including hearing, touch and nociception [15, 16, 17, 18, 19]. *PIEZO* channel openings lead to membrane depolarization and Ca^2+^ influx, which can trigger intracellular Ca^2+^ signaling cascades and thereby regulate diverse physiological processes. Accordingly, *PIEZO*‐mediated mechanosensation and mechanotransduction play important roles in enabling organisms to perceive their environment, with direct consequences for survival. Despite the essential roles of *PIEZO* in mechanical sensing, it remains largely unexplored whether natural genomic variations in *PIEZO* exist and how such variations contribute to organismal adaptation to high ‐altitude.

Here, we assembled a high‐quality chromosome‐level locust genome for the migratory locust from the QTP, and investigated TE evolutionary trajectories across macroevolutionary and microevolutionary scales. We analyzed population genomics of locusts sampled along an altitudinal gradient ranging from 2 to 4100 m. We characterized TIP landscapes and identified TE dynamics associated with natural selection. The results revealed a prominent expansion of some TE subfamilies (PiggyBac, Helitron, TcMar, CR1 and RTE) in the locust genome, and concomitantly an abundance of adaptive sweeps associated with these TEs. Furthermore, *PiggyBac* insertion into the *PIEZO* gene led to alternative splicing and enhanced downstream AMPK signaling, thereby improving flight performance of Tibetan locusts under hypoxic conditions. Our results reveal the significant contribution of TEs to genome evolution and environmental adaptation and reconcile micro‐ and macroevolution of TE dynamics for adaptation at the superfamily level.

## 2 Results

### 2.1 TEs Drove Genome Expansion and Shape Genome Architecture in Orthoptera

We generated a high‐quality genome assembly for the migratory locust from a single male individual collected in the QTP (Tables S1 and S2 and Figure S1). Genome‐wide synteny analyses indicated a strong syntenic relationship between our assembly and the recently published assemblies [7, 8] (Figure S2).

Comparison of gene features across nine insect species showed that the two species from the order Orthoptera (*L. migratoria* and *Schistocerca gregaria*) possessed significantly longer genes compared to insect species from other orders (Figure S3A). Gene size in Orthoptera was mainly contributed by longer intron (Figure S3B,C), due to abundant TE insertions in intronic regions: 86.16% of introns overlap with TEs, and 73.56% of intron length is attributable to TE presence (Figure S3D). Additionally, 15.09% genes (2851 out of 18 899) possessed at least one exon having high TE level (defined as those regions with average signal density ≥ 0.8 in the heatmap) (Figure S3D and Table S3).

Thirteen representative insect species from seven orders exhibited great variation in genome size (Figure S4A, middle panel). Likewise, TE contents also showed great variation across these species, ranging from 11.60% to 72.13% in TE percentage of the whole genome (Figure S4A, right panel). Total TE content was significantly correlated with genome size (Pearson's r = 0.9958, *p* = 3.38e^−13^) (Figure S4B), while no significant correlation was detected between coding sequence size and genome size, suggesting that TE content contributes substantially to genome size evolution across these species.

**FIGURE 1:**
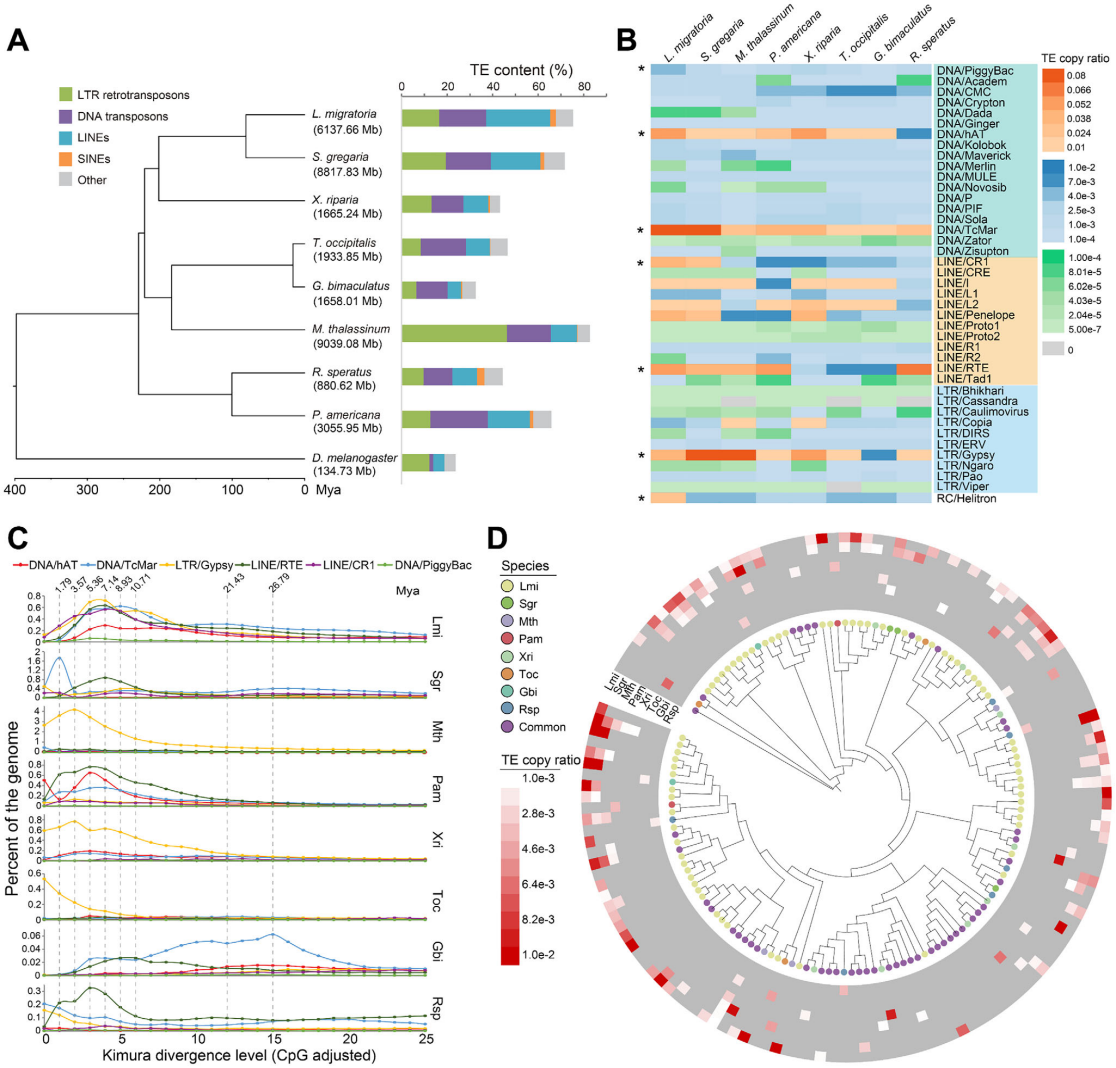
The content and dynamics of TEs in genome evolution across the migratory locust and closely related species. (A) Phylogeny of representative species in Orthoptera and Blattodea and the distribution of different TE types in these species. (B) Heatmap showing the dynamics of copy numbers of various TE superfamilies across eight species. The TE superfamilies were roughly classified into three major classes, i.e., DNA transposon, LINE, and LTR, each highlighted in different background colors. (C) Landscape of Kimura divergence level of six representative TE subtypes in eight insect species. The dotted line indicates the putative burst time points of each TE subtype. TE burst time was estimated based on the CpG‐adjusted Kimura substitution levels and the nucleotide substitution rate of 2.8 × 10^−9^ per site per generation reported previously. The Kimura divergence level estimates the number of nucleotide substitutions per site, and is expressed as percentage divergence between a TE copy and the consensus sequence. (D) Phylogenetic tree of common and species‐specific PiggyBac consensus sequences across eight insect species. The outer heatmap shows the ratio of different PiggyBac families among eight species. Species name abbreviations: Lmi, *Locusta migratoria*; Sgr, *Schistocerca gregaria*; Mth, *Meconema thalassinum*; Pam, *Periplaneta americana*; Xri, *Xya riparia*; Toc, *Teleogryllus occipitalis*; Gbi, *Gryllus bimaculatus*; Rsp, *Reticulitermes speratus*.

We then investigated the diversification of genome size and genome architecture in six representative insect species in the order Orthoptera, i.e., *Gryllus bimaculatus*, *Xya riparia*, *Teleogryllus occipitalis*, *L. migratoria*, *Schistocerca gregaria*, *Meconema thalassinum*, and two species from the order Blattodea, *Reticulitermes speratus* and *Periplaneta americana*. The genome sizes varied substantially among these eight species, and presented a gradient variation between 881 to 9039 Mb, thus providing a good basis for studying genome size evolution. The composition of four major TE types differed substantially among these species (Figure 1A), with LTR‐RTs, DNA transposons, and LINEs representing top three contributors to genome size in these species. *L. migratoria*, *S. gregaria*, and *M. thalassinum* possessed more intact LTR‐RTs (Figure S5A,B), suggesting the higher activity of LTR‐RTs in the three species. However, SINEs only account for 2.47% in the locust genome.

Correlation analysis showed that the genome size was most highly correlated with DNA transposons (R = 0.97, *p* = 2.5e^−5^), followed by LINEs (R = 0.93, *p* = 3.5e^−4^), LTRs (R = 0.85, *p* = 3.6e^−3^), and SINEs (R = 0.53, *p* = 0.14) (Figure S6A). LINE/RTE (R = 0.95, *p* = 3.6e^−4^), LTR/Gypsy (R = 0.76, *p* = 0.029), and DNA/TcMar (R = 0.75, *p* = 0.033) represented the top three TE superfamilies showing high correlation with genome size (Figure S6B–D).

We next explored the dynamics of TE superfamily copy numbers across the eight species. We compared the copy ratio of different TE superfamilies in these species. Different TE superfamilies exhibited substantial variation in copy number among the eight species (Figure 1B). On one hand, there were remarkable differences in copy number with multiple orders of magnitude across TE superfamilies. For instance, the copy ratios of different TE superfamilies to the total TEs in a genome range from the lowest LTR/Viper (Lmi: 6.01 × 10^−8^) to the highest DNA/TcMar (Lmi: 9.0 × 10^−2^). On the other hand, substantial interspecific differences in copy number were observed for several TE superfamilies. Apparently, the top three most abundant TE superfamilies in *L. migratoria*, *S. gregaria*, and *M. thalassinum* were DNA/TcMar, LTR/Gypsy, and LINE/RTE respectively (Figure 1B). Both DNA/PiggyBac (4.73 × 10^−3^ vs 5.74 × 10^−4^‐3.12 × 10^−3^) and RC/Helitron (2.94 × 10^−2^ vs. 2.11 × 10^−3^‐6.68 × 10^−3^) exhibited higher copy percentages in *L. migratoria* than in the other seven species (Figure 1B).

Additionally, we evaluated the expansion status of representative TE superfamilies based on the putative model that TE copies sustainably increased during evolution. We observed the expansion of copies of distinct TE superfamilies in five extant species, with the exception of *R. speratus*, *G. bimaculatus*, and *X. riparia*. After the order Orthoptera diverged from Blattodea, copies of three superfamilies (LINE/RTE, DNA/TcMar, and LTR/Gypsy) were elevated in Blattodea, while copies of DNA/hAT and LINE/CR1 were uniquely increased in Orthoptera. Notably, *M. thalassinum* exhibited the highest copy number change, of which the majority (72%) was contributed by the LTR/Gypsy superfamily. Additionally, the common ancestor of *L. migratoria* and *S. gregaria* (ancestor 1) showed the secondary highest copy number change, encompassing all six TE superfamilies (Figure S7 and Tables S4 and S5), with DNA/TcMar and LINE/RTE occupying the highest proportion. After the divergence of *L. migratoria* and *S. gregaria*, the LTR/Gypsy, DNA/TcMar, and LINE/CR1 superfamilies were expanded in *S. gregaria* in copy number, while the remaining three TE superfamilies were enlarged in *L. migratoria*. To sum up, four TE superfamilies (DNA/TcMar, LINE/RTE, LINE/CR1, and LTR/Gypsy) are major contributors to the expansion of genome size in *L. migratoria* and *S. gregaria*.

Besides, we analyzed the divergence landscape (based on Kimura substitution) of representative TE superfamilies across eight species (Figure 1C). Specifically, the DNA/hAT superfamily showed a small burst around 7.14 Mya in *L. migratoria*, while this superfamily exhibited a strong burst in *P. americana* and a weak burst in *X. riparia* at approximately 5.36 Mya. The burst time of the DNA/TcMar superfamily varied considerably, with the most ancestral burst time at 8.93 Mya for *L. migratoria*, followed by 7.14 Mya for *P. americana*, and 1.79 Mya for *S. gregaria*. Notably, the DNA/TcMar superfamily in *G. bimaculatus* underwent two rounds of bursts (26.79 and 5.36 Mya). The LTR/Gypsy superfamily showed two small peaks (7.14 and 10.71 Mya) in *L. migratoria*, while *M. thalassinum* and *X. riparia* shared the recent burst time of LTR/Gypsy at 3.57 Mya. Additionally, an insertion peak for the DNA/PiggyBac superfamily was observed at Kimura distance of 3 in *L. migratoria* (Figure 1C), while no peak was detected for other species. Altogether, the burst and accumulation of TE superfamilies during evolution could correspond to their copies (Figure 1B), partially explaining their copy number difference.

To investigate the evolutionary relationships of six major TE superfamilies (e.g., LTR/Gypsy, DNA/hAT, DNA/TcMar, DNA/PiggyBac, LINE/CR1, and LINE/ RTE) across these species, we performed phylogenetic analyses based on their respective consensus sequences (Figure 1D and Figure S7). For clarity, only families with an average copy number >10 across the eight species were retained for downstream analyses. For the DNA/PiggyBac superfamily, in addition to the PiggyBac consensus sequences shared with RepBase (93 families, 65.49%), the majority of species‐specific PiggyBac elements (22 families, 15.49%) originated from *L. migratoria*, highlighting the exceptional diversity of PiggyBac transposons in the locust genome. Consistently, across all six TE superfamilies, a substantial proportion of consensus sequences exhibited markedly higher copy numbers in *L. migratoria* than in the other seven insect species (Figure S8).

Taken together, DNA/TcMar, LTR/Gypsy, DNA/PiggyBac, and LINE/RTE showed high activity across species and were highly correlated with genome size variation, suggesting the impact of TEs on insect genome evolution. Prominently, DNA/PiggyBac represents a TE superfamily that was uniquely expanded in the locust genome, implying its potential contribution to locust genomic evolution.

### 2.2 Genetic Divergence and Genome‐Wide Signatures for QTP Adaptation in Locusts

**FIGURE 2:**
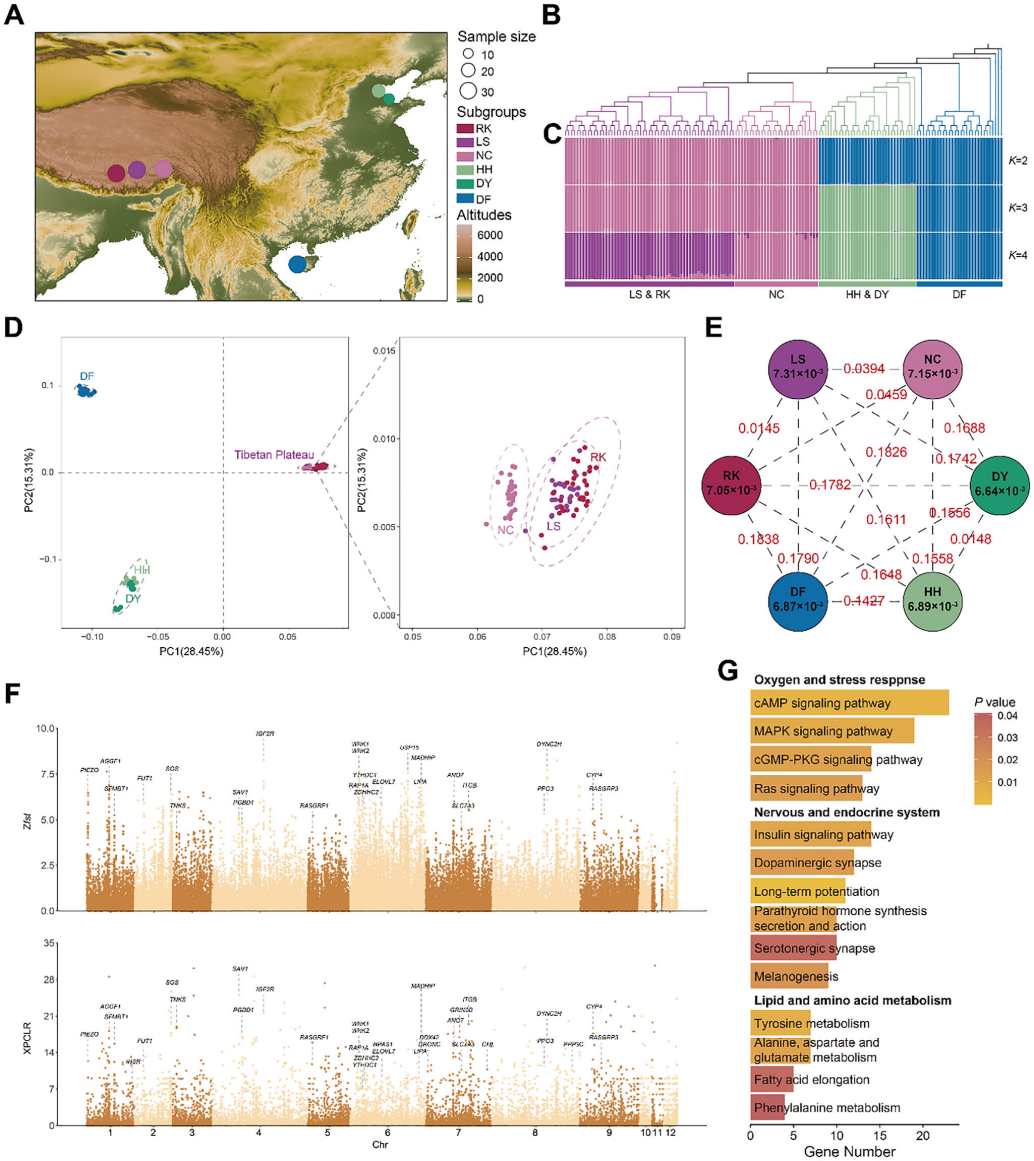
Population structure and genetic divergence analyses of locust from six geographically distinct locations. (A) Geographic distribution of 153 locust samples collected for whole‐genome resequencing. The world map was constructed using the R package ggplot2 with the worldclim dataset (https://worldclim.org/). (B) A neighbor‐joining tree of all the wild locust samples. Branch colors denote populations. (C) Genetic structure analysis with different numbers of ancestry kinship (*K*  =  2, 3, and 4). Locust from LS&RK, NC, DY&HH, and DF are indicated by the colored bar at the bottom. (D) Principal Component Analysis (PCA) of the locust populations. Left: PCA of 153 locust accessions. The proportions of variance explained by the principal components are presented in the axis labels. Right: PCA of the plateau populations. (E) Summary of nucleotide diversity (π, in circles) and population divergence (*fst*, between lines) among different geographic populations. (F) Manhattan plot of the Z*fst* values and XP‐CLR values (y‐axis) in windows of 100 kilobases (kb) using a 50 kb slide across all autosomes (X‐axis). Names of genes within the highest peaks are shown. For a full list of the linked genes, see Tables S10 and S11. (G) GO enrichment of candidate genes. The windows with the top 5% Z*fst* and XP‐CLR simultaneously were considered as candidate selective regions, and genes in these regions as candidate genes. For details of the GO entry, see Table S13.

To reveal the elevational genetic divergence and environmental adaptability of QTP in locust populations, we performed whole‐genome resequencing for 153 individuals from six distinct geographical locations in China spanning 2 to 4,100 m in latitude (Figure 2A; Table S6). A total of 16.55 Tb of Illumina short‐read sequencing data were obtained with the average sequencing depth of each individual ∼ 18 × (Table S7). We identified a total of 152 113 496 high‐quality single‐nucleotide polymorphisms (SNPs) (Table S9), among which 72.53% of these SNPs in intergenic regions and 0.23% in coding regions (Figure S9B). In addition, the distribution of SNPs varied across chromosomes, with a higher density in chromosome 5 (2.91%) and a lower density in chromosome 12 (1.31%) (Figure S9A and Table S9). SNP density was positively correlated with chromosome length and linkage disequilibrium (LD), but negatively correlated with gene density (Figure S10). The results indicate that chromosome‐scale variation in genome architecture and recombination landscapes contributes to the heterogeneous distribution of SNPs across chromosomes.

To explore population structure, we first reconstructed the neighbor‐joining (NJ) tree including all individuals. Phylogenetic reconstruction of all populations sampled were divided into three clusters: the QTP group (i.e., LS, RK and NC), the Southern plain group (i.e., DF) and the Northern plain group (i.e., DY and HH), which further supports the presence of three sub‐populations (Figure 2B). Northern plain populations are ancestrally closely related to QTP populations compared with the southern plain populations. To inferences of the genetic structure, a Bayesian model‐based approach was implemented. When *K* = 2, the sampled locusts were genetically divided into the QTP populations and the plain populations; when *K* = 3, the Northern plain populations were separated from the plain populations; when *K* = 4, population LS and RK of QTP, population NC of QTP, and the Northern plain populations formed three additional genetic clusters (Figure 2C). A principal component analysis (PCA) based on whole genome SNPs provided further corroborating evidence for these groupings (Figure 2D). Our results provided evidence that locusts from QTP, Northern China and Southern China are genetically distinct.

To identify population variation, nucleotide diversity and fixation index were calculated. Compared with the Southern plain populations (DF, *f_st_* = 0.1790‐0.1838), the Northern plain populations (DY and HH, *f_st_* = 0.1558‐0.1782) presented a lower level of genetic differentiation from QTP populations (LS, RK and NC) (Figure 2E). The low genetic differentiation was found for population LS vs. population RK (*f_st_* = 0.0145), as well as population DY vs. population HH (*f_st_* = 0.0148). Moreover, populations LS&RK and populations HH&DY were considered as representative plateau populations and plain populations, respectively. Elevated genetic diversity (Wilcoxon rank sum test, *p* = 0.0022) and Tajima's D (Wilcoxon rank sum test, *p* < 2.2e^−16^) were observed in the plateau populations, implicating the presence of cryptic population structure or demographic forces (Figure S11).

We next analyzed the demographic history of the four altitudinal populations (Figure S12). The results demonstrated that no substantial overall divergence in historical population size exists between plateau and plain populations. During the period spanning 1.1 Ma to 0.1 Ma, demographic trajectories of effective population size (Ne) were largely congruent across the four examined populations. Population sizes peaked around 1 Ma and subsequently underwent demographic contraction. Approaching 0.1 Ma, plain populations (DY/HH) slightly deviated in demographic trends from their plateau counterparts and exhibited greater demographic stability. The decoupled population dynamics between plateau and plain lineages potentially linked to the population split occurring at approximately 90 Kya [11] and the Last Glacial Maximum (26.5–19 Kya).

We further identified candidate genomic regions under selection potentially associated with high‐altitude adaptation by comparing plateau populations (LS&RK) with plain populations (HH&DY). We used three selection statistics, *Fst*, XP‐CLR, and nSL. Z*fst* (Z‐transformed *Fst*) identifies genomic regions that exhibit high variation in allelic frequency between groups. XP‐CLR (the cross‐population composite likelihood score) method detects selective sweep by comparing allele frequencies between two populations [20]. nSL is a haplotype‐based statistic detecting both soft and hard sweeps in population genomic data from a single population [21]. By employing a combination of three selection analyses, we identified a total of 731 candidate selected genes within the top 5% of statistical values and 57 genes within the top 1% (Figure 2F; Figure S13 and Tables S10–S12).

Functional enrichment analyses revealed that these 731 selected genes were involved in oxygen and stress response, nervous and endocrine system, and lipid and amino acid metabolisms (Figure 2G; Table S13). Several pathways, represented by the important selected genes such as *INSR*, *CALM*, and *CAMK4*, could contribute to the oxygen and stress response in high altitude through cAMP signaling, MAPK signaling, cGMP‐PKG signaling, and Ras signaling. The nervous and endocrine system, represented by selected genes *MITF*, *PPP3CB*, and *PLCB1*, could be involved in the insulin signaling pathway, dopaminergic synapse, serotonergic synapse, and melanogenesis. Some selected genes such as *FASN* and *HSD17B12* are important for lipid and amino acid metabolisms and could facilitate adaptation through tyrosine metabolism, fatty acid elongation, phenylalanine metabolism, and alanine, aspartate, and glutamate metabolism. In addition to these enriched pathways, some positively selected genes, such as *SIRT1*, *IGF2R*, *CAT*, *DNAH*, and *CDT1*, were documented to potentially functionally important for locusts’ plateau adaption Among the top 1% selected genes, *PIEZO*, *LIPA*, and *HSD17B12* were identified in pathways involved in oxygen and stress response, or nervous and endocrine system regulation.

### 2.3 TEs Mediate Population Divergence and Selective Sweeps Associated with QTP Adaptation

**FIGURE 3:**
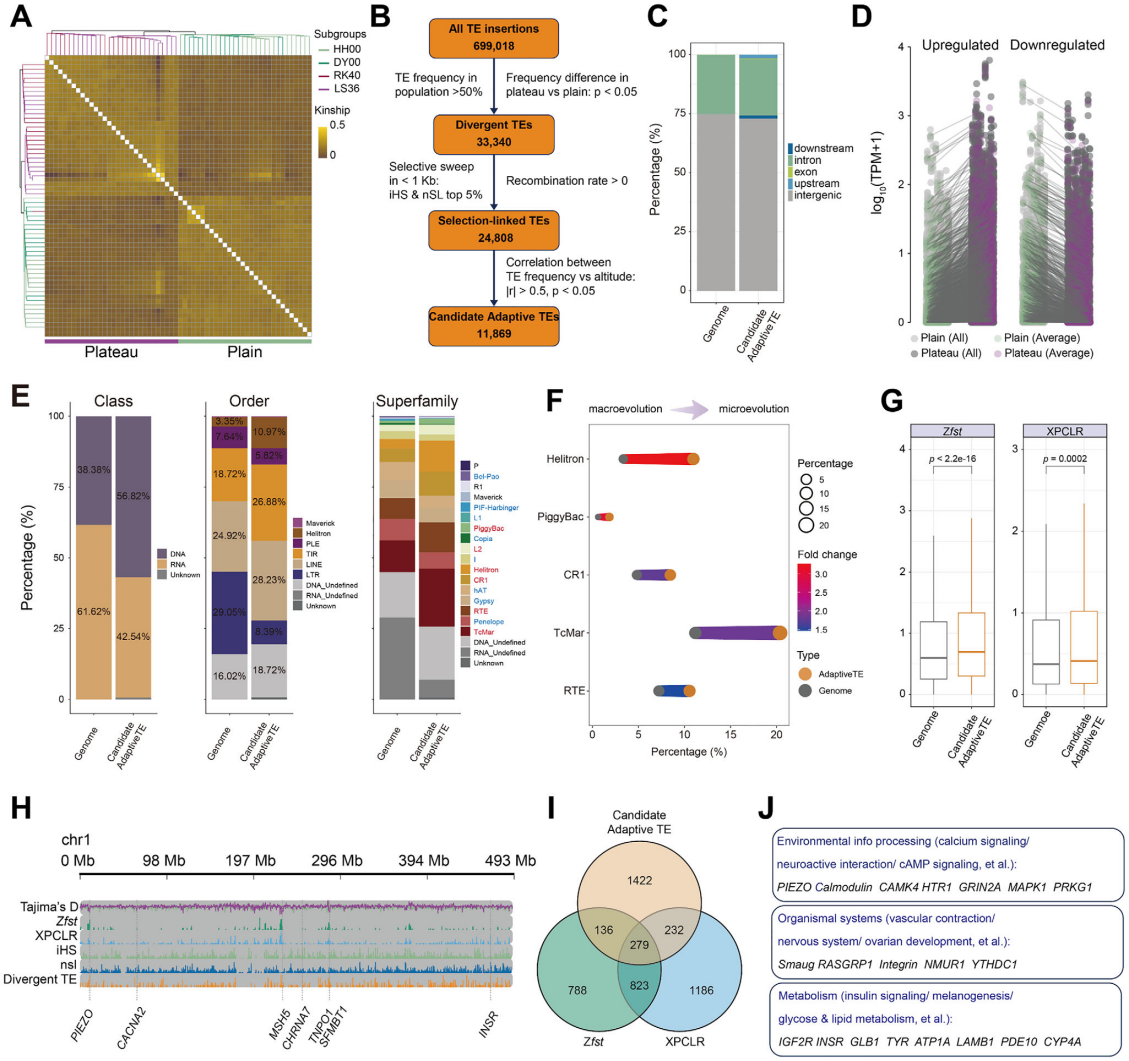
TE insertions reshape population genomic landscape and mediate selective sweeps associated with latitude adaptation. (A) Kinship matrix of the locusts from four natural populations based on whole‐genome TE insertion polymorphisms (TIPs). (B) Workflow for detecting candidate adaptive TIPs in the locust populations. (C) Percentage of candidate adaptive TEs and genome in different genomic regions. (D) Expression of candidate adaptive TE‐influenced genes in plain and plateau populations. Upregulation (left panel) and downregulation (right panel) of gene expression are shown. (E) The percentage of different types of TEs in the reference genome and candidate adaptive TEs set. The TE types marked in red or blue in the legend indicate significant differences between the genome and the candidate adaptive TE sets, with red denoting expansion and blue denoting contraction within the candidate adaptive TE sets. (F) TE superfamilies contributing to both locust genome evolution and population differentiation. This visualization highlights TE superfamilies that have undergone lineage‐specific expansion at the macroevolutionary scale and are concurrently involved in population‐level adaptive divergence. Pie size represents the proportion of all the TEs of a TE superfamily in the whole reference genome (grey) or in the candidate adaptive TE set (orange) The color of the connecting lines indicates the fold change in the above percentage of TEs of a TE superfamily in the candidate adaptive TE set compared with that in the genomic background. (G) Selection evaluation using fst value and XP‐CLR score in the candidate adaptive TE insertion regions (n = 11 869) and the genome‐wide region (n = 122 000). Significance levels of difference were calculated using Wilcoxon rank‐sum test. (H) The landscape of genomic selection in chr1. Tajima's D, Z*fst*, and XP‐CLR plotted using 100 kb windows and 50 kb slide. The iHS and nSL number represents the number of significant sites (value > P95) screened by the iHS or nSL method within a 100 kb window. (I) Number of candidate genes under selective sweep identified by the three methods listed in each of the Venn diagram components. Three datasets of candidate genes are shown in Tables S19 and S20. (J) Important high‐altitude adaptation pathways and genes through enrichment analysis of those 279 selected genes.

We next investigated the potential contribution of TEs to population differentiation in the locust. A total of 699 018 TE insertions polymorphisms (TIPs) were identified across representative plateau (LS&RK) and plain (HH&DY) populations (Figure S14A). Although the overall number of TE insertions did not differ significantly between populations (Figure S14B), TIP‐based analyses could clearly separate plateau and plain populations (Figure 3A; Figure S14C). These results indicate that TEs are associated with genetic differentiation among locust populations across different altitudes.

To identify candidate adaptive TE insertions, we performed an integrated analysis to look for evidence of positive selection associated with TEs using both TIPs and SNPs neighboring the TEs (see Methods). First, we selected those TIPs whose frequency in lowland or plateau populations is more than 50%. Furthermore, the TE insertion frequency should be significantly diverged between lowland or plateau populations (Chi‐square test, p <0.05). With these criteria, we identified 33 340 divergent TEs (Figure 3B). Second, we analyzed signatures of selective sweep in the regions flanking the TEs using SNPs alleles as a proxy. We used two different haplotype‐based statistic methods: iHS [22] and nSL [21]. We considered a SNP to be significant when both iHS and nSL values were above 95th percentile (see Methods). We then looked for TE insertions located within 1 kb from the significant SNP. Furthermore, we only consider as candidate TEs those present in regions with recombination rates >0. With these principles, we identified 24 808 TEs linked with selective sweep (Table S14). Last, we performed correlation analysis between insertion frequency and altitude of TEs in the five locust populations (Pearson correlation coefficients |r|> 0.5, p < 0.05)). Using this framework, we identified a total of 11 869 TEs and thereafter referred to as candidate adaptive TEs (Figure 3B).

We next examined LD patterns surrounding candidate adaptive TEs by calculating r^2^ values and comparing them across different distances flanking TE insertion sites. We observed that LD gradually decays with increasing distance from candidate adaptive TEs while LD surrounding the associated SNPs remained relatively constant. The results supporting the notion that many adaptive TE insertions are closely associated with the underlying selective signals and are likely to play causal roles in adaptive evolution (Figure S15).

These candidate adaptive TEs reside and potentially affect 2069 genes (Figure 3B; Table S15). Candidate adaptive TEs were approximately evenly distributed across all autosomes but were markedly underrepresented on the sex chromosomes (chr6) (Figure S16A,B and Table S16). Most adaptive TEs (72.88%) were located in intergenic regions, whereas 2.97% were inserted into putative regulatory regions (defined as 5 kb upstream or downstream of a gene), and 23.92% were located within intronic (Figure 3C).

To evaluate the regulatory potential of the candidate adaptive TEs, we screened these TEs for transcription factor binding sites (TFBSs). A total of 682 388 TFBSs were identified across these candidate adaptive TEs, with an average of 57.5 TFBSs per TE (Figure S17). These results indicate that a substantial proportion of candidate adaptive TEs harbor putative regulatory elements and may contribute to gene regulatory variation. We further investigated whether candidate adaptive TEs are potentially important sources for gene expression variation. We defined differentially expressed genes (DEGs) as those differing in their basal expression in four adult tissues between plateau and plain locust subpopulations, or those significantly induced by two plateau stress, i.e., ultra violet radiation and hypoxia (Table S8). We thus identified 7371 DEGs. Among those genes harboring candidate adaptive TEs, 48.64% of them were DEGs (Figure S18). In short, candidate adaptive TE insertions are associated with upregulation of 510 genes and downregulation of 475 genes in plateau populations. The greater distance was observed between a candidate adaptive TE and a gene, the weaker its impact on gene expression changes (Figure 3D; Figure S19).

We next assessed whether TEs co‐localize and link with genic regions under selection or population divergence. We overlapped the adaptive TE insertion sites with the selective sweep regions detected by XP‐CLR and the divergent regions detected by Z*fst*. Our results showed that the adaptive TE insertion regions were strongly overlapped with the selected genic loci. Compared with the whole genomic regions harboring TEs, the genomic regions harboring the candidate adaptive TEs exhibited significantly higher fixation index (Wilcoxon rank sum test, *p* < 2.2e^−16^) as well as in the XP‐CLR value (*p* < 0.001) (Figure 3G). It is worth noting that as the candidate adaptive TE frequency difference between plateau and plain populations increases, the degree of selection and population differentiation also increases (Figure S20). The results further implied that these candidate adaptive TE insertions were involved in genomic changes underlying natural selection processes.

To explore the evolutionary dynamics of candidate adaptive TE subfamilies, we compared two TE pools: genome‐wide TE set and the candidate adaptive TE set at the class, order, and superfamily levels. At the superfamily level, fold changes in copy number between the candidate adaptive TE set and the reference genome ranged from −9.74 to 3.27 (Tables S17 and S18). Fourteen of the 17 annotated TE superfamilies showed significant differences in abundance between the candidate adaptive TE set and the reference genome. Notably, six TE superfamilies (Helitron, TcMar, CR1, RTE, PiggyBac, and L2), together accounting for 55.68% of candidate adaptive TEs, were significantly enriched, indicating copy number expansion. Specifically, Helitron represented 10.97% of the candidate adaptive TE set, a 3.27‐fold increase compared with its proportion in the locust genome (3.35%) (χ^2^ test, p < 2.2 × 10^−16^), whereas PiggyBac accounted for 1.85% of candidate adaptive TEs, a 3.01‐fold increase relative to the genome‐wide proportion (0.62%) (χ^2^ test, p < 2.2 × 10^−16^) (Figure 3E; Tables S17 and S18). Notably, five of these TE superfamilies also exhibited high copy numbers and pronounced copy number expansion at the macroevolutionary scale (Figure 3F). Together, these results indicate that TE copy number expansion is broadly consistent across both microevolutionary and macroevolutionary scales in insects.

Finally, we focused on the TE‐associated adaptive gene. An integrated landscape of genome‐wide selection was constructed by combining Z*fst*, XPCLR, iHS, nSL, and TE frequency. These different selection metrics revealed largely concordant patterns of selection signatures (Figure 3H; Figure S21). In total, 279 genes were co‐selected across three methods (XPCLR, Z*fst* and association with candidate adaptive TEs) (Figure 3I; Figure S22 and Tables S19 and S20). These genes were mainly enriched in pathways related to signal transduction (e.g., cAMP signaling pathway, cGMP‐PKG signaling pathway, Ras signaling pathway and calcium signaling pathway), endocrine system (e.g., insulin signaling pathway and melanogenesis), digestive system (e.g., protein digestion and absorption and gastric acid secretion), nervous system (e.g., long‐term potentiation, glutamatergic synapse and serotonergic synapse). Notably, the greatest number of enriched gene set (12 of 279) was assigned to the cAMP signaling pathway (Figure 3J).

**FIGURE 4:**
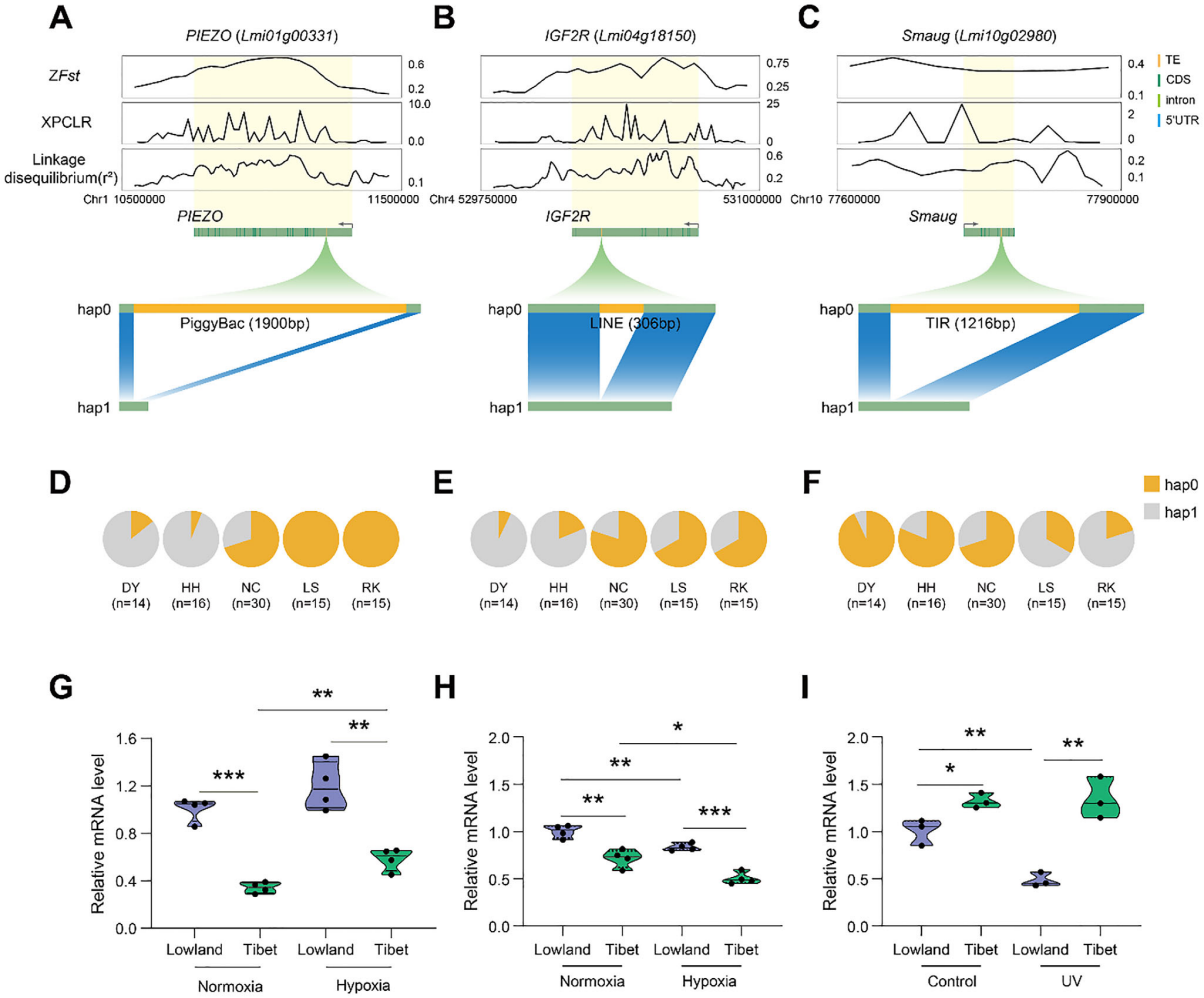
Transposon insertions altered gene expression and facilitated altitude adaptation. (A–C) Selective sweep signal and TE‐haplotypes of *PIEZO*, *IGF2R* and *Smaug*. Hap0: present TE insertion; Hap1: absent TE insertion. (D‐F) Frequencies of TE‐haplotypes of *PIEZO*, *IGF2R* and *Smaug* in DY, HH, NC, LS, RK population. (G) *PIEZO* expression in adult flight muscle in response to hypoxia in Tibetan and lowland locusts (n = 4). (H) IGF2R expression in adult flight muscle in response to hypoxia in Tibetan and lowland locusts. Hypoxia treatment was 10 Kpa for 48 h (n = 4). (I) *Smaug* expression in adult flight muscle in response to UV in Tibetan and lowland locusts UV treatment for 14 h (n = 4). Student's t test: ^*^ *p* < 0.05, ^**^ *p* < 0.01, ^***^ *p* < 0.001. The values are mean ± SD.

To validate these candidates, we selected 3 genes (*PIEZO*, *Smaug* and *IGF2R*) from the above functional categories based on their selection signal, expression variation and potential functional significance. The results showed that all three genes exhibited strong selection signals represented by Z*fst*, XP‐CLR and LD test (Figure 4A–C). The three genes harbored TE insertion polymorphisms (TIPs) (Figure 4D–F). Adults were subjected to hypoxia stress by exposing to 10 kPa oxygen partial pressure for 48 h, or to UV stress by exposing to 2.6 W/m^2^ radiation for 14 h. The three genes were significantly responsive to either UV or hypoxia stress (Figure 4G–I). In particular, *PIEZO* expression in flight muscle revealed markedly lower levels in Tibet locusts under both hypoxia (49.8% reduction, Student's t test, *p* = 0.005) and normoxia conditions (39.6% reduction, Student's t test, *p* < 0.001) compared with lowland locusts (Figure 4G).

### 2.4 PiggyBac‐Mediated *PIEZO* Mutations in Tibet Locusts

**FIGURE 5:**
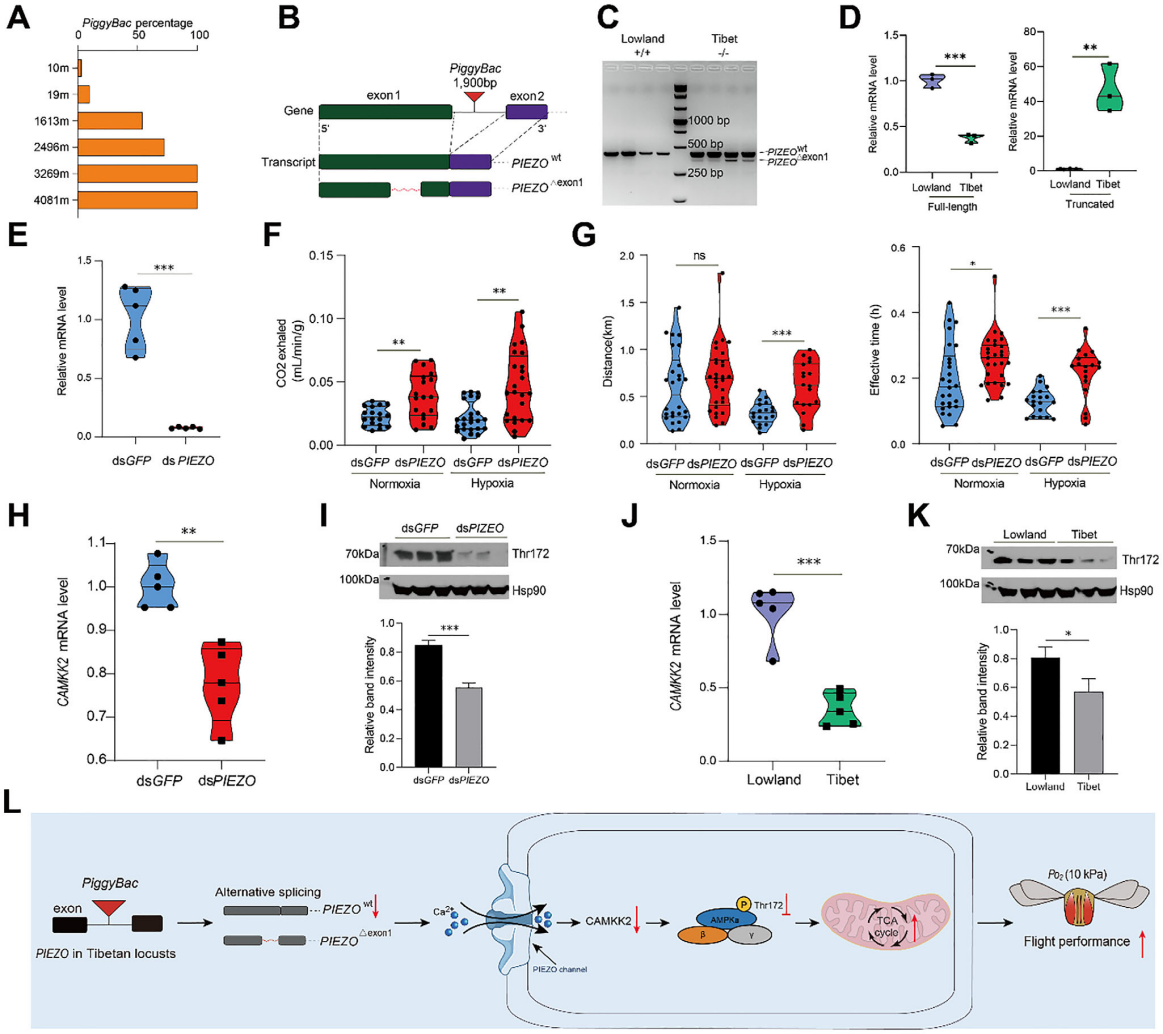
*PiggyBac* insertion into *PIEZO* causes alternative splicing, and *PIEZO* promotes hypoxic flight through AMPK signaling in Tibetan locusts. (A) Detection of *PiggyBac* insertion at different altitudes (10 m, n = 31; 19 m, n = 20; 1613 m, n = 24; 2496 m, n = 11; 3269 m, n = 26; 4081 m, n = 30). (B) Gene and transcript structure of *PIEZO* with *PiggyBac* insertional mutation. Red block means a 69bp‐deletion in *PIEZO* ^Δexon1^ mutant. Only the first three exons are shown. *PIEZO* ^wt^ means full‐length transcript, and *PIEZO* ^Δexon1^ means the truncated transcript. (C) Gel image showing *PIEZO* alternative transcripts caused by *PiggyBac* insertion. (D) Expression of full‐length and truncated transcripts of *PIEZO* in lowland and Tibet locusts (n ≥ 3). (E) Expression knockdown of *PIEZO* via dsRNA injections in adult flight muscle. ds*GFP* was used as control (n = 5). (F) CO_2_ production rate of locusts under normoxia and hypoxia conditions. Hypoxia condition was 10 kPa; n ≥ 18. (G) Effects of *PIEZO* expression knockdown on flight performance of locusts under normoxia and hypoxia conditions. Flight distance and effective time were measured using flight mill system. n ≥ 18. (H) *PIEZO* knockdown abolished *CAMKK2* expression (n = 5). (I) Western blot revealed decreased phosphorylation level of AMPKα after *PIEZO* interference. Thr172 represents phosphorylation of AMPKα. Left, representative blots. Right, quantification of Thr172/Hsp90 (n = 3). (J) *CAMKK2* expression in Tibet and lowland locusts (n = 5). (K) Western blot revealed decreased phosphorylation level of AMPKα in Tibetan locusts. Left, representative blots. Right, quantification of Thr172/Hsp90 (n = 3). (L) A working model for an adaptive role of *PIEZO* mutations mediated by a *PiggyBac* insertion. The TE insertion into *PIEZO* intron in the Tibetan locusts causes *PIEZO* alternative splicing that represses *CAMKK2* expression and AMPKα phosphorylation, consequently activating glucose and lipid metabolisms in flight muscle, and finally promotes flight performance of locusts under hypoxia. ^*^ *p* < 0.05, ^**^ *p* < 0.01, ^***^ *p* < 0.001 by Student’ s *t* test for subfigures D‐E and H‐K, a Mann‐Whitney U test for subfigures F‐G. The values are mean ± SD.

Among the 279 candidate genes, *PIEZO* (present as a single paralog in the locust genome, Figure S23A) exhibited the most prominent signal of selection (Z*fst*, XP‐CLR and LD values; Figure 4A,D,G). Genome‐wide selection analysis revealed a strong selective sweep at *PIEZO* locus between plateau and plain populations (Figure 4A; Figure S24). Sanger sequencing validated the presence of 1900‐bp DNA transposon PiggyBac inserted into the fist intron of *PIEZO*. PCR‐based genotyping further showed that the frequency of PiggyBac decreased with decreasing altitude, from fixation as homozygous insertions in plateau population to only 5.88% insertion in lowland populations (Figure 5A; Table S21). These results suggest that PiggyBac insertion may contribute to population‐specific regulation of *PIEZO* expression.

To determine whether PiggyBac insertion mediates expression variation of *PIEZO*, we subsequently examined *PIEZO* expression in plateau and lowland populations. Transcript splicing analysis of *PIEZO* revealed that there occurred a 69‐bp deletion in exon 1 in the plateau locusts with the PiggyBac insertion into *PIEZO* (Figure 5B; Figure S23B). PCR and Sanger sequencing confirmed that, in Tibetan locusts, due to the insertion of PiggyBac, there are two distinct transcripts of *PIEZO*, while in lowland locusts, there is only one single transcript (Figure 5C; Figure S23C). Using flight muscle tissue from individual ancestral (i.e., absent of PiggyBac insertion) and derived (i.e., with presence of PiggyBac insertion) locusts, transcript‐specific PCR and sequencing further validated two alternative *PIEZO* transcripts: a canonical full‐length transcript and a truncated isoform lacking the 69‐bp region (Figure S23D,E). Thus, genotyping of genomic DNA and cDNA from ancestral and derived individuals all demonstrated that the alternative splicing event coincided with the PiggyBac insertion.

Transcript‐specific expression analysis showed that the canonical transcript was significantly less abundant in Tibetan locusts than in lowland locusts. Although expressed at low levels, the truncated transcript was nonetheless more abundant in Tibetan than in lowland locusts (Figure 5D). Expression analysis of individual locusts with the specific genotypes demonstrated the similar results (Figure S23D,E). These findings indicate that the PiggyBac insertion in the first intron of *PIEZO* induces alternative splicing, leading to reduced expression of the canonical transcript. Quantification of total *PIEZO* expression in Tibet and lowland locusts corroborated the findings (Figure 4G).

### 2.5 *PIEZO* Promotes Hypoxia Adaptation in Tibet Locusts through AMPK Pathway

In high‐altitude adaptation research, the concurrent measurement of flight and respiratory parameters under both hypoxia and normoxia conditions enables the differentiation between *PIEZO*‐mediated basal metabolic regulation and its hypoxia‐driven adaptive phenotypes. ds*PIEZO* injection resulted in 86% decrease for *PIEZO* mRNA in flight muscles of locusts (Students’ t test, *p* < 0.001) (Figure 5E). Measurements of CO_2_ production revealed that *PIEZO* knockdown significantly elevated the respiratory rate in adults under normoxia (*p* = 0.002) and, more markedly, by approximately 48% under hypoxia (*p* < 0.001) (Figure 5F). Flight assessment further demonstrated that adults with repressed *PIEZO* expression fly longer distances under hypoxia (*p* < 0.001) and showed prolonged effective flight durations under both normoxia (*p* = 0.012) and hypoxia (*p* < 0.001) (Figure 5G). These findings demonstrate that *PIEZO* suppression enhances adult respiratory metabolism and promotes flight capacity under hypoxic conditions.

To elucidate the detailed regulatory mechanisms by which *PIEZO*, we performed transcriptome profiling and metabolomic profiling of flight muscle in *PIEZO* knockdown and ds*GFP* treated locusts. 547 DEGs were significantly enriched into multiple biological pathways (Figure S25B,C). Pathway enrichment analysis highlighted the AMPK signaling pathway. Consistent with this result, *PIEZO* knockdown significantly reduced CAMKK2 expression (*p* = 0.001, Students’ t test), which in turn led to a marked decrease in the phosphorylation levels of AMPKα at Thr172 (Figure 5H,I). Our study showed that *PIEZO* knockdown significantly repressed *CAMKK2* expression, possibly by regulating calcium ion entry into cells. *CAMKK2* repression then substantially reduced phosphorylation levels of Thr172 on AMPKα. Metabolomic profiling following *PIEZO* interference further revealed significant up‐regulation of metabolic pathways associated with glycolipid metabolism, including the tricarboxylic acid (TCA) cycle (Figure S25D). All together, these results uncovered a potential working pathway in which *PIEZO* mediates AMPK signaling via CAMKK2 ‐dependent phosphorylation of AMPKα. Through this pathway, *PIEZO* knockdown enhances glucose and lipid metabolic activity in flight muscle, thereby contributing to improved flight performance under hypoxic conditions.

Finally, we examined whether the CAMKK2–AMPKα signaling axis differed between plateau and lowland locust populations. Consistent with the *PIEZO* knockdown experiments, plateau locusts exhibited significantly lower CAMKK2 expression (*p* < 0.001) and reduced phosphorylation levels of AMPKα (Figure 5J,K).

Collectively, our results indicated that the PiggyBac insertion within *PIEZO*, which predominantly occurs in plateau locusts is associated with alternative splicing and reduced *PIEZO* expression. This regulatory change modulates CAMKK2‐AMPKα signaling, resulting in enhanced glucose and lipid metabolism in flight muscle and ultimately conferring improved locust flight performance under plateau hypoxic conditions (Figure 5L).

## 3 Discussion

The current and other genome assemblies of the locust all highlighted the massive accumulation of TEs in their genomes [6, 7, 8]. Our results underscore the pivotal role of TEs in shaping genome structural evolution. More importantly, this study reveals that TEs exert pervasive effects on gene function, thereby promoting adaptive evolution and, in turn, facilitating their own persistence and expansion across genomes over evolutionary timescales.

The expansion of the genome is primarily attributed to its high repetitive elements (75.33%), with TEs as the predominant component. Multiple studies have explored the relationship between TE content and genome size [23, 24]. In our study, genome size shows a strong positive correlation with the content of different TE types, such as DNA transposons, LINEs and LTRs, while a weak correlation with the content of SINEs (Figure S6), which might be partially due to the small proportion of SINEs in insect genome. Therefore, TEs contribute significantly to genome size evolution, consistent with the previous findings.

We observed that the genes of two Orthoptera insects (*L. migratoria* and *S. gregaria*) were significantly longer than those in other insect species from different orders. Further analysis indicated that the longer genes in these two species were primarily attributed to the longer introns but not the exons, which was also supported by the presence of abundant TEs in intronic regions, which is coincident with the previous study [6, 23, 25]. Together, TE expansion has played a role in shaping both gene length and overall genome size of the migratory locust, and more broadly across the superfamily Acridoidea.

The relative contributions of different TE types to genome size can vary markedly across taxonomic groups. For example, DNA transposons and LINE retrotransposons were the primary contributors to genome size expansion in Coleoptera insects [26]. By contrast, in *Heliconius* butterflies (Lepidoptera), SINE elements accounted for a large proportion of TEs and were the major drivers of genome size variation [26]. At the superfamily level, LINE/RTE, LTR/Gypsy, and DNA/TcMar represented top three TE superfamilies exhibiting the strongest correlations with genome size. DNA/hAT, LINE/RTE, and DNA/PiggyBac showed significant expansion in *L. migratoria*. DNA/TcMar, DNA/hAT, LINE/CR1, LINE/RTE, and LTR/Gypsy represented five TE superfamilies with high copy number in Orthoptera insects and great variations across these species. By contrast, DNA/PiggyBac harbored relatively lower absolute copy number across these insect genomes compared with other TE superfamilies. However, it shows a high amplitude of change across these insect species. The copy number of DNA/PiggyBac was the highest in *L. migratoria* compared with other insects (including *S. gregaria* and *M. thalassinum* with greater genome size). Such a unique expansion of PiggyBac elements in *L. migratoria* suggests their crucial roles in evolution and adaptation of the migratory locust.

*PiggyBac* is a typical Class II DNA transposon that mobilizes via a cut‐and‐paste mechanism, noted for its high transposition efficiency, precise excision, and minimal genomic footprint. It has now been identified across diverse eukaryotic lineages, including most mammals [27, 28]. In the locust genome, PiggyBac superfamily contains 19 934 copies, which were significantly higher than in other orthopteran genomes. Phylogenetic analysis indicated that the PiggyBac superfamily was composed of 72 families in *L. migratoria*, of which the majority showed prominent expansion in the locust genome. Additionally, the locust harbored the most unique PiggyBac families (22, 15.49%) relative to other insects (Figure 1). Notably, the burst of *PiggyBac* insertion in the locust genome occurred recently (around Kimura distance of 3), which might suggest their ongoing activity in shaping the genomic architecture. Moreover, TIPs analysis based on population resequencing data showed that *PiggyBac* insertions were highly dynamic, suggesting that their persistent roles in the regulation of gene expression, splicing patterns, and important traits.

Different from SNPs, TE insertions may be retained by natural selection not only through directly altering the expression or function of key genes [1], but also by inducing local genomic instability that facilitates the emergence of new SNPs. Some of these SNPs can further modify gene function and, under environmental selection, become fixed [9]. This process leads to the co‐occurring phenomenon between TIP and SNP [5]. Therefore, combining TE and SNP analysis can help to reveal more deeply the genetic variation of populations and their evolutionary patterns. This study proposes and implements an analytical approach and comprehensive framework that integrates TE and SNP variation information to analyze population genetic landscape and adaptive evolutionary mechanisms. This framework not only provides a new perspective for population genomics research, but also opens up new avenues for elucidating TE‐driven adaptive evolutionary processes.

This study demonstrated that TIPs can serve as an effective genetic marker to distinguish plateau from lowland populations, indicating that large numbers of unique TE insertions have been retained in different groups. Analysis of demographic history and directional selection along altitude confirmed that the genetic divergence based on TIPs is less likely caused by differences in effective population size. Theoretically, TE insertions should occur randomly, and thus the proportion of each superfamily in the candidate adaptive TE set would be expected to mirror their overall genomic representation. However, we observed fold changes ranging from −9.74 to 3.27 among different superfamilies, suggesting that certain types of TEs, such as Helitron, PiggyBac, and TcMar, are more likely to drive within‐species differentiation. Notably, the TEs involved in intra‐ and inter‐species divergence appear to be interconnected. For example, the lineage‐specific expansion of *PiggyBac* elements in the locust genome not only contributes to genome evolution at the species level but also plays a major role in population‐level differentiation. This phenomenon highlights that TE variants that become gradually fixed and spread within populations can ultimately shape lineage divergence and even interspecific differences. In other words, the macroevolution could be essentially the cumulative outcome of microevolutionary processes. Hence, TEs provide molecular evidence across evolutionary scales: in the short term, TE insertions continuously generate functional genetic diversity within populations, thereby driving adaptive responses to environmental pressures such as high‐altitude; over longer timescales, TEs have shaped fundamental genomic features, including chromosomal rearrangements, genome size expansion, and the evolution of repetitive landscapes among species. Thus, TEs are not only a bridge linking population‐level adaptation and species‐level divergence but also a major force driving the formation of biodiversity.

The candidate adaptive TEs exhibit strong preference of insertion location first to intergenic region (72.88%), then the intronic region (23.92%), and regulatory region within 5 kb downstream or upstream of a gene (2.97%). The insertion preference is very consistent between the adaptive TEs and whole‐genome TEs (Figure 3B). Regulatory regions of a gene can extend from proximal region next to the gene to hundreds of kb downstream or upstream of the gene or through 3D genome structure [29]. Insertion into these regions could cause widespread regulatory mutations that alter expression of local or nearby genes. Meanwhile, TE insertion can affect focal genes from long distance through rewiring the regulatory networks or high‐order chromatin structure. In addition, TE insertions could be associated with genetic variation at nearby genes through linkage with selective sweeps that decay over long distance [30, 31]. Therefore, TE insertions may represent one major source of gene expression variation and regulatory evolution.

TE‐mediated selective sweep may contribute to locust hypoxia adaptation on the QTP. A total of 279 positively selected genes with adaptive TE insertions were identified. Some pathways enriched in these genes have been reported to be involved in organism hypoxia adaptation and several biological processes, such as cAMP signaling pathway [32, 33], insulin signaling pathway [34, 35], cGMP‐PKG signaling pathway [36, 37]. For example, mutations in the *PTPN1* gene contribute to hypoxia adaptation by modulating insulin signaling [11]. The gene enrichment also suggests that the cAMP signaling pathway play a key role in plateau (e.g., hypoxia) adaptation of locust.

As one of the most prominent candidate adaptive TE gene, *PIEZO* encodes a mechanosensitive ion channel component that resides in the cell membrane, regulating cellular mechanotransduction by inducing Ca^2+^ influx in response to extracellular stimuli [14, 38]. *PIEZO* could play an important regulatory role in controlling respiration and mediating flight capacity in insects. Our study showed that locusts with *PIEZO* knockdown exhibited enhanced respiration efficiency and flight ability, especially under ambient hypoxia conditions. Previous studies showed that *PIEZO* also influences mechanosensation of respiration and blood pressure and cardiovascular functions [18, 19, 39]. These functions could work in cascade or with associated mechanisms that are regulated by *PIEZO*.

This study further unveiled the downstream regulatory pathway of *PIEZO*, in which *PIEZO* regulates MAPK signaling and energy metabolism in flight muscle. The α‐subunit of AMPK can be directly phosphorylated at Thr172 by the calcium‐sensitive kinase CAMKK2, also known as CAMKKβ, in response to calcium influx [33, 35, 40, 41, 42], thus linking calcium signaling to AMPK regulation of energy metabolism [43, 44]. AMPKα activation promoted the TCA cycle and energy metabolism [45]. Previous findings showed that the *PIEZO1* channel stimulates ATP production by enhancing mitochondrial respiration and glycolysis in vascular endothelial cells [46]. Therefore, *PIEZO* regulates CAMKK2‐AMPK pathway that controls energy homeostasis in flight muscle, possibly through controlling intracellular calcium influx. This functional pathway likely underlies a broader physiological mechanism by which insects adapt to low oxygen stress through modulation of basic metabolic processes. Previous studies have shown that Tibetan *L. migratoria* exhibit less inhibition of metabolic processes like the TCA cycle, which is advantageous for aerobic metabolism and ATP production under extreme hypoxia, resulting in higher respiratory efficiency without damaging mitochondrial morphology [10, 47]. This is consistent with our results. CAMKK2 has also been shown to activate AMPK under hypoxia [48]. The patterns of CAMKK2 and Thr172 observed at high altitudes are consistent with *PIEZO* interference, leading us to hypothesize that the hypoxic environment at high altitudes activates CAMKK2, which in turn affects AMPK and enhances aerobic metabolism in high‐altitude *L. migratoria*, improving their flight capacity. These results indicate that the *PIEZO* gene plays a critical role in behavioral adaptations associated with energy metabolic homeostasis.

The observation that a TE insertion into the first intron of *PIEZO* causes the truncation of the first exon and alternative splicing represents a classic molecular paradigm of TE‐mediated genome‐wide transcriptional plasticity [49]. The observation can be explained at least by two main non‐exclusive pathways. First, at the transcript level, the inserted element introduces a powerful competitive intronic splice acceptor that pairs with an otherwise dormant, cryptic 5' splice donor located inside the first exon; this results in the spliceosome cutting out the terminal region of the first exon. During pre‐mRNA processing, the spliceosome bypasses the canonical boundary in favor of this internal cryptic site, leading to the splicing out of the terminal portion of the first exon and generating a partially truncated mature transcript alongside alternative isoforms [50, 51]. Second, at the genomic level, the initial physical insertion of the TE can trigger localized, imprecise DNA repair or micro‐deletions that structurally slice off the end of the first exon and its canonical splice junction [52, 53]. These overlapping molecular pathways demonstrate the profound capacity of TEs to rewire regulatory networks.

Finally, our study delivers a comprehensive genomic resource, including a high‐quality chromosome‐level reference genome and the largest resequencing dataset of locusts to date. We generated genome‐wide maps of SNP variation and TE insertion polymorphisms, offering a broad catalog of genetic variation. In addition, transcriptomic datasets were produced from multiple tissues, populations across altitudinal gradients, and locusts exposed to hypoxia and ultraviolet stress. These resources substantially expand the genomic landscape of the migratory locust and establish a robust foundation for future research into insect genome evolution and environmental adaptation.

## 4 Materials and Methods

### 4.1 Sample Collection and DNA Extraction

The locust used for *de novo* genome assembly was originally collected from Lhasa on the QTP. For PacBio sequencing, the genomic DNA (an average size of 40 kb) was extracted from the hindlegs of an individual male adult. The remaining tissues of the male adult were used to prepared the Hi‐C libraries.

Individuals of *L. migratoria* were sampled from 6 geographically distinct locations in China spanning 2 to4,100 m in latitude: Dongfang, Hainan province (DF, 2 m above sea level), Huanghua, Hebei province (HH, 4 m), Dongying, Shandong province (DY, 3 m), Nyingchi, Tibet (NC, 2943–3060m), Lhasa, Tibet (LS, 3554–3620 m), and Rikaze, Tibet (RK, 3981–4100m). For whole‐Genome sequencing, 153 individuals from 6 locations (30 from DF, 20 from HH, 14 from DY, 30 from NC, 30 from LS, and 29 from RK) were randomly selected for DNA extraction with the MiniBEST Universal DNA Extraction Kit (Takara Bio Inc., Dalian, China) (Table S6).

### 4.2 Rearing

Locusts were maintained in well‐ventilated cages (50 × 50 × 50 cm^3^) with a stocking density of 100 newly hatched larvae per cage. The environmental conditions were controlled to maintain a temperature of 28 ± 2°C, approximately 55% air humidity, and a light cycle of 14 h light (L):10 h dark (D). The locusts were fed fresh wheat seedlings twice daily. Adult male locusts on the fifth day of emergence were selected for experiment.

### 4.3 Genome Survey

To estimate the genome size, heterozygosity, and repeat content, we employed Jellyfish v2.2.0 [54] for generating a 21 *k‐mer* frequency distribution. The error rate was defined as the depth of *k‐mer* = 1. The genome size was estimated using the formula: Genome size = (*k‐mer* count / main peak depth) × (1 – Error rate). GCE v1.0.2 [55] was used for estimating the heterozygosity ratio and repeat content ratio.

### 4.4 PacBio Library Construction and Sequencing

Each SMRTbell library was constructed using the Pacific Biosciences SMRTbell express template prep kit 2.0 following the manufacturer's instructions. The constructed libraries underwent size selection with a BluePippin system for molecules 20 Kb, followed by primer annealing and the binding of SMRTbell templates to polymerases with the DNA/Polymerase Binding Kit. After primer annealing, the SMRTbell library was sequenced on the Pacific Bioscience Sequel II platform by Annoroad Gene Technology Company (Beijing, China).

### 4.5 *De novo* Genome Assembly and Evaluation

PacBio Sequel II reads were used for constructing the primary assembly. The PacBio long reads were corrected using Canu v1.8 [50]. The primary contigs were assembled using the Canu v1.8 [50] pipeline based on the corrected subreads. Two rounds of corrections were performed for the assembly using Illumina short reads by Pilon tool v1.23 [51] with default parameters. To evaluate the genome quality, the Benchmarking Universal Single‐Copy Orthologs (BUSCO, v5.2.2) [52] with insecta_odb10 database was employed to assess the assembly quality and the gene annotation with genome and protein modes, respectively.

### 4.6 Chromosome‐Level Pseudomolecule Scaffolding

The Illumina clean paired‐end reads yielded from one Hi‐C library were mapped to contigs using BWA‐MEM (v0.7.17) [53]. The assembled contigs were scaffolded onto chromosome pseudomolecules through sorting, orientation, and ordering using 3DDNA [56] with default parameters. The scaffolded pseudo‐chromosomes yielded by 3DDNA were visually checked using the JuicerBox [57] and manually corrected. Juicer [58] was employed to generate a final version of the *L. migratoria* genome assembly, which was comprised of twelve chromosomes.

### 4.7 Repeat Annotation

To identify repeat sequences across the whole genome, we integrated ab initio and homology‐based methods for annotation of TEs in the locust. For the *de novo* search, RepeatModeler (v1.0.11) (https://github.com/Dfam‐consortium/RepeatModeler) was used for constructing a species‐specific TE library. Additionally, LTR_FINDER v1.05 [59] and LTRharvest v1.5.11 [60] were employed for identifying full‐length LTR retrotransposons (LTR‐RTs), and LTR_retriever v1.9 [61] was used to remove the inaccurate identification results to generate a non‐redundant LTR‐RT library. Then, repeat sequences were identified using RepeatMasker v4.1.1 based on a *de novo* library through concatenating the species‐specific TE library and the LTR library. For the homology‐based searches, RepeatMasker was performed to identify repeat elements based on a known repeat library (RepBase 15.02) (https://www.girinst.org/server/RepBase/index.php). Tandem repeat sequences were identified using the Tandem Repeat Finder (TRF v4.07b) [62].

### 4.8 Gene Prediction and Functional Annotation

To predict protein‐coding genes in the locust, we employed a strategy combining de novo prediction, homology‐based prediction, and transcriptome‐based prediction. For ab initio prediction, the PASApipeline v2.3 [63] was used to predict gene structures from the transcripts assembled by Trinity v2.12 [64]. The inferred gene structures were used to train a gene model using Augustus v3.2.3 [65]. For homology‐based prediction, proteins of several insect species from public databases were mapped to the locust genome using GenomeThreader v1.7.3 [66]. For transcriptome‐based prediction, RNA‐seq data were mapped to the locust genome using HISAT2 v2.2.1 [67], and transcripts were assembled using StringTie v2.1.6 [68]. The candidate ORFs (open reading frames) in the assembled transcripts were estimated using TransDecoder v5.1.0 (https://github.com/TransDecoder/TransDecoder). Subsequently, EVidenceModeler [63] was used to integrate the prediction results from different sources with weights for different methods as follows: StringTie > PASA > GenomeThreader > Augustus. A final gene set was generated after eliminating the gene models with weak supporting evidence.

Protein‐coding genes were functionally annotated by search against known databases using BLAST, such as SwissProt and NCBI non‐redundant protein database. InterProScan v5.32 [69] was employed for assignment of gene ontology (GO) terms and conserved Pfam domains. KEGG orthology (KO) terms were assigned to all genes through search against Kofam database using KofamScan [70] with default parameters.

### 4.9 Gene Family and Phylogenomic Analysis

To reconstruct the evolutionary history of *L. migratoria*, we selected twelve representative insect species, including six holometabolous insects (i.e., *Apis mellifera* [RefSeq: GCF_003254395.2], *Tribolium castaneum* [Ensembl Metazoa: Tcas5.2], *Bombyx mori* [Ensembl Metazoa: ASM15162v1], *Papilio xuthus* [RefSeq: GCF_000836235.1], *Anopheles gambiae* [Ensembl Metazoa: AgamP4], and *Drosophila melanogaster* [Ensembl Metazoa: BDGP6]), and six hemimetabolous insects (i.e., *Bemisia tabaci* [RefSeq: GCF_001854935.1], *Acyrthosiphon pisum* [RefSeq: GCF_005508785.1], *Blattella germanica* [GenBank: GCA_003018175.1], *Schistocerca gregaria* [RefSeq: GCF_023897955.1], *Schistocerca cancellata* [RefSeq: GCF_023864275.1], and *Gryllus bimaculatus* [http://gbimaculatusgenome.rc.fas.harvard.edu]). For each species, the splice isoforms were removed to retain only the longest transcripts. Orthologous groups (OGs) across these thirteen species were obtained via clustering using OrthoFinder v2.5.4 [71] with default parameters. To construct the phylogenetic tree of *L. migratoria* and other twelve species, 1285 single‐copy orthologous genes were collected and aligned using MUSCLE v3.8 [72]. Then, these aligned sequences were merged to generate a super alignment matrix. The amino acid substitution model selection was conducted using ProtTest v3.4.2 [73] based on the Bayesian Information Criterion (BIC) score, and the best‐fit model was determined as ‘LG+I+G+F’. Subsequently, a maximum likelihood phylogenetic tree was constructed using RAxML v8.2.10 [74] with 500 bootstrap replications. We employed r8s [75] to estimate the divergence time between species or clade. Additionally, six known calibration points obtained from TimeTree (http://timetree.org/), such as 238.5–307.2 million years ago (Mya) for *D. melanogaster* vs. *A. mellifera*, 238–345 Mya for *D. melanogaster* vs. *T. castaneum*, 250 Mya for *D. melanogaster* vs. *A. gambiae*, 221–341 Mya for *L. migratoria* vs. *G. bimaculatus*, 80–157 Mya for *B. mori* vs. *P. xuthus*, and 255–360 Mya for *L. migratoria* vs. *B. germanica*, were employed for calibrating divergence time. FigTree (v1.4.3) was used for the visualization of the species tree.

### 4.10 Gene Family Evolution Analysis

The expansion and contraction of orthologous groups were identified using computational analysis of gene family evolution (CAFÉ v4.2.1) [76] based on the difference in gene number within each orthologous groups of each species. To determine the significance for expansion and contraction of OGs, the *P*‐value was calculated for each OG using a Monte Carlo resampling procedure, and the significance threshold was set as *p*‐value < 0.05.

### 4.11 Correlation Analysis between TE Types and Genome Size

To explore the correlation between different TE types and genome size, we performed Pearson correlation analyses using *cor.test* function in R between TE size and genome size at different levels. The correlation plot between TE size and genome size was generated using ggplot2.

### 4.12 Copy Number Change of Six TE Superfamilies in the Phylogeny

To reveal the copy number change of six representative TE superfamilies in the phylogeny, we adopted a putative model that the copy number of all TE types sustainably increased along the phylogeny. The expansion status of TE superfamilies within each species was evaluated through comparison against the common ancestor.

### 4.13 Divergence Analysis of TEs

To estimate the divergence level of TEs across difference species, we performed a divergence analysis of the TE superfamilies based on Kimura 2‐parameter distances. Based on the alignment files after genome masking, the Kimura distances between genome copies and TE consensus sequence were calculated using three scripts embedded in the util directory of the RepeatMasker package (http://www.repeatmasker.org), i.e., buildSummary.pl, calcDivergenceFromAlign.pl, and createRepeatLandscape.pl. The insertion time (T) of TE superfamilies were estimated using the formula T = *K*/2r, *K* denotes the Kimura distance, r denotes the mutation rate. Here, we adopted a nucleotide substitution rate of 2.8 × 10^−9^ per site per generation previously reported [77]. Thus, the Kimura divergence level estimates the number of nucleotide substitutions per site, and is expressed as percentage divergence between a TE copy and the consensus sequence. Generally, the Kimura divergence level ranges from 0% to 50% [78]. Low values (0%–5%) indicate recently inserted elements, intermediate values (5%–15%) indicate moderately old elements, and high values (>15%) indicate ancient insertions [79].

### 4.14 Phylogenetic Analysis of the TE Superfamilies

To infer the evolutionary relationship of six superfamilies (DNA/hAT, DNA/TcMar, DNA/PiggyBac, LTR/Gypsy, LINE/CR1, and LINE/RTE) across eight species, we performed a phylogenetic analysis of TE consensus sequences. The consensus sequences of TEs were aligned using MUSCLE [80] with default parameters. IQtree [81] was first used to select the best model, and the maximum likelihood tree was constructed using IQtree [81] based on the optimal model with 1000 ultrafast bootstrap replicates.

### 4.15 SNP Calling

First, raw reads were filtered using the fastp program (v.0.12.4) [82] with default parameters, and aligned to the locust reference genome using BWA (v.0.7.17‐r1198) [53] with the command ‘mem’. SAMtools (v.1.7) [83] was then used to convert the format of SAM files, sort BAM files, and filter mapping quality with the ‘‐q 30’ parameter. The Genome Analysis Toolkit (GATK, v.4.2.0.0) [84] modules MarkDuplicates were used to make duplicates, and HaplotypeCaller was run on each bam file in a genomic variant call format (GVCF) mode. The GVCF files from 153 accessions were consolidated into a single GVCF file, from which SNPs and small indels were identified using a joint calling approach. The SNPs were further filtered using the following criteria: 1) VariantFiltration modules with ‘‐filter‐expression QD < 2.0 || MQ < 40.0 || FS > 60.0 || MQRankSum < −12.5 || ReadPosRankSum < −8.0’ parameter. 2) variants with missing rate of >20% or a minor allele frequency (MAF) of <0.05 were removed using vcftools (v.0.1.17) [85]. The whole set of variants was annotated using SnpEff (v.4.3t) [86] with default parameters.

### 4.16 Population Structure Analysis

To construct the population‐based phylogenetic tree, we utilized vcf2phylip (v2.7) (https://github.com/edgardomortiz/vcf2phylip) to convert the VCF file into PHYLIP format and reconstructed using RAxML software (v8.2.12) [74] with a 100‐times bootstrap under the GTRGAMMA substitution model. The output tree was plotted in iTOL [87]. Population structure was analyzed by the model‐based clustering method ADMIXTURE (v1.3.0) [88], with cluster numbers (*K*) ranging from 2 to 4. For PCA, we used the PLINK (v1.9) [89] software to convert the VCF files into PLINK files. Then, PCA was carried out using the PLINK (v1.9) [89] software with default parameters.

### 4.17 Demographic History

Changes in Ne of each group was inferred using a hidden Markov model approach as implemented in pairwise sequentially Markovian coalescence (PSMC, https://github.com/lh3/psmc) with parameter as follows: ‐N30 ‐t15 ‐r5 and ‐p ‘4+25*2+4+6’. Time was measured in units of 2N_0_ generations, and the Ne at time *t* was scaled to N_0_. The neutral mutation rate *µ* was used to infer N_0_ and scale the TMRCA (time to the most recent common ancestor) and N_e_ values into chronological time. The mean generation time *g* was set at 0.5 year, and *µ* was estimated as 0.1 × 10^−8^.

### 4.18 Genetic Diversity

Nucleotide diversity (π) within subgroups and fixation statistics (*fst*) between different subgroups were calculated by vcftools (v.0.1.17) [85]. Tajima’ s D values of each group (DY & HH, LS & RK) were calculated using vcftools (v.0.1.17) [85] with 100‐kb sliding windows and a step size of 50 kb.

### 4.19 Selective Sweeps Detection

The XP‐CLR score were calculated using the XP‐CLR package [20] and *fst* were calculated using the vcftools (v.0.1.17) [85] with 100‐kb sliding windows and a step size of 50 kb. The *fst* were Z‐transformed to obtain Z*fst*. The windows with the top 5% Z*fst* and the top 5% XP‐CLR simultaneously were selected as candidate selective regions, and genes in these regions were considered as candidate genes.

### 4.20 iHS and nSL Analyses

To create the phased panel, genotypes were placed onto phased SNP haplotypes using Beagle [90]. The iHS and nSL statistics were calculated using the selscan (v2.0.0) [91] software and phased SNP haplotypes data with the default parameters. After obtaining results from each statistic, we normalized them using the norm (v1.3.0) package in 10 frequency bins across each chromosome. We considered iHS and nSL normalized values to be statistically significant for a given SNP if they were greater than the 95th percentile of the distribution of normalized values, and these SNPs are considered significant SNPs.

### 4.21 Identification of TIPs and Candidate Adaptive TE in Locusts

We generated a locust TE database by combining the *de novo* annotation results from RepeatMasker and the RepBase database. Using the ngs_te_mapper2 software [92], we identified the distribution of TE in each sample of the groups by integrating resequencing data, the reference genome, and the locust TE database. For each sample, sequencing reads were analyzed using the command “ngs_te_mapper2 ‐o sp1_output ‐f sp1.fastq ‐r genome.fasta ‐l library.fasta”, the insertion calls from all samples were subsequently merged, and a genome‐wide matrix of transposable element insertion polymorphisms (TIPs) was generated for downstream population genomic analyses. Kinship was calculated using GCTA software [93] and PCA were calculated using PLINK (v1.9) [89] based on TIPs. Classification of transposons according to Wicker et al. [94].

We selected candidate adaptive TE based on the principles as follows: 1) TE frequency in plateau or plain populations is >50%, and the frequency of TE insertion significantly differs between plateau and plain populations (Chi‐square test, p < 0.05). If a TE is adaptive, we expect its frequency to increase in the population, even for a TE present in high recombination region [95]. 2) There is a significant correlation between TE insertion frequency and altitudes of locust populations (Pearson correlation coefficients |r|> 0.5, p < 0.05). This correlation analysis is used to infer directional selection for TE insertions along the elevational gradient in the five populations, i.e., HH, DY, NC, LS, and Ri populations (Figure 2). 3) Signals of selective sweeps in the vicinity of the candidate TE insertions are detected using two different haplotype‐based statistics, i.e., iHS [22] and nSL [21]. Many studies demonstrated that the regions flanking adaptive TEs show reduced nucleotide diversity compared with the neutral expectations, thus displaying signatures of (partial) selective sweeps [9, 95, 96, 97]. The iHS test mainly detects events of hard sweeps. The nSL test detects both soft and hard sweeps, most notably in the cases of sweeps from standing variation and incomplete sweeps, and is more robust to recombination rate variation [21]. We defined a SNP to have a significant iHS or nSL values when, after normalizing by frequency and chromosome location, the normalized values were above 95th percentile of the distribution of values for SNPs. We then looked for candidate TE insertions located within 1 kb from the significant SNP. 4) TE is inserted in a high recombination region of the genome, i.e., recombination rate > 0. TEs located in regions with high recombination rates are less likely to have increased in frequency neutrally compared with TEs located in low recombination regions [95].

### 4.22 Identification of Transcription Factor Binding Sites within Candidate Adaptive TEs

Transcription factor binding site (TFBS) sequences from the model organism Drosophila melanogaster were downloaded from the JASPAR database (https://jaspar.elixir.no/). Candidate adaptive TE sequences were scanned against the TFBS motifs using FIMO software [98], and the predicted TFBS information for each candidate adaptive TE was subsequently obtained.

### 4.23 Enrichment Analysis

GO annotation terms for locust genes were generated through the geneontology (https://www.geneontology.org/), and KEGG annotation terms were generated through the KAAS (https://www.genome.jp/tools/kaas/). KEGG and GO enrichment analysis of the sweep genes was carried out using R package clusterProfiler (v.4.12.6) [99]. Enrichment significance was analyzed with Fisher's exact test.

### 4.24 RNA‐Seq Analysis

Total RNA was isolated form four tissues (brain, flight muscle, and ovariolar tip) in plateau and plain locust samples under three different conditions (UV, hypoxia, and normal) (Table S8). Three separate differential expression analyses were performed: (1) altitude‐related: between plateau and plain locust subpopulations, (2) UV‐responsive: between control and UV‐treated samples, and (3) oxygen‐responsive: between normoxic and hypoxic samples. RNA‐seq libraries were constructed and sequenced on an Illumina. The clean reads were mapped against the locust genome using hisat2 (v2.2.1) software [67]. SAMtools (v.1.7) [83] was then used to convert the format of SAM files, sort BAM files, and make index. Stringtie (v.2.1.6) [100] was used to calculated FPKM values for each gene. Analysis of differential gene expression between plateau and plain, normoxia and hypoxia, or UV and normal light was performed using the DESeq2 R package (v.1.44.0) [101]. Genes with an adjusted *P value* < 0.05 and | log_2_FoldChange| > 1 found were assigned as differentially expressed.

Double‐stranded RNA (dsRNA) was injected into male adult locusts on the fifth day of emergence, and the flight muscles were collected 48 h post‐injection, ensuring five independent replicates of five locusts for each group. Total RNA was extracted using the RNA extraction kit from Promega (Beijing, China). cDNA libraries were prepared following the Illumina protocol. Raw sequence reads were filtered using SOAPnuke to eliminate adapter sequences and low‐quality reads, resulting in clean reads that were subsequently aligned to the locust genome using HISAT software. Differentially expressed genes (DEGs) were determined using DESeq2 software, with genes exhibiting an adjusted *p* value < 0.05 classified as differentially expressed. KEGG enrichment analysis was performed with KOBAS software to identify relevant biological pathways, employing Fisher's exact test for significance evaluation.

### 4.25 RNA Interference (RNAi)

Primers were designed using the Primer 5 software, and the RNA interference vector was constructed with the pEASY‐Blunt Zero Cloning Kit. Double‐stranded RNA (dsRNA) was synthesized using the T7 RiboMAX Express RNAi system (Promega, USA). Ten µg of dsRNA was injected into the third ventral part of the abdomen of male adult locusts on the fifth day of emergence. Gene expression levels were assessed 48 h post‐injection using qRT‐PCR, with the *dsPIEZO*‐injected locusts serving as the target gene knockdown group and the *dsGFP*‐injected locusts as the control. The primers for *PIEZO* are listed in Table S22.

### 4.26 Hypoxic and UV Treatment

Hypoxic treatment was performed using a portable tri‐gas incubator (Smartor 118; Huayi Ningchuang Intelligent Technology Co. Ltd, Zhejiang, China) in which the air and nitrogen supply and partial pressure of oxygen (*pO2*) [BC3.1] could be precisely controlled. Specifically, air was blown into the hypoxic chamber, and balanced with pure nitrogen to achieve the required *pO2* level, i.e., 10 kPa for hypoxia and 21 kPa for normal air. To maintain specific environmental conditions, the incubator was placed at a temperature of 28 ± 1°C and a photoperiod of 14:10 h (L:D). Ten male locusts that had emerged for 5 days were placed into an incubator treated for 48 h for the experiment. Three replicates of 10 adults were used for each treatment.

A UVB radiation dose of 2.6 W/m^2^ at wavelength 302 nm, which is the daily maximum solar radiation in Lhasa, Tibet, was selected for the UVB treatment. Three‐day‐old female adult were exposed to UVB for 14 h daily from 7:00 AM. The control group and the untreated hours of the UVB treated groups were maintained under normal light conditions in the incubator. The incubator was controlled under a temperature of 28 ± 1°C and a photoperiod of 14:10 h (L:D). Three replicates of 10 adults were used for each treatment.

### 4.27 Behavioral Measurements

The CO_2_ production rate was measured as follows: locusts were weighed and recorded before being placed individually in respiratory tubes of a closed system for assessment. The multi‐channel insect respiratory metabolic system was activated for measurement. After the insect respiration rate stabilized, data collection commenced using the ExpeData software (Sable Systems International, Las Vegas, NV, USA) [102, 103]. The MUX FLOW MULTIPLEXER was adjusted to digital mode (e.g., Digital 01–08). The CO_2_ production rate was calculated as the volume of CO_2_ generated per gram of body weight per minute.

For measurement of flight behavior of locusts, an insect flight mill method was used as in previous studies [102, 104]. The horizontal arms of the flight mill were constructed from 1.5 mm plastic rods, extending a radius of 12 cm, with each rotation covering approximately 75 cm. Fans were positioned at the top of each flight mill to sustain flight at a wind speed of 1.5 m/s. FlightMill (Pengcheng Electronic Technology Center, Beijing, China) software was used to record flight parameters, including total flight distance and effective flight duration. During the flight ability assessment, adult male locusts were allowed to fly on the flight mill for a duration of 60 min.

### 4.28 Metabolomics Sequencing and Analysis

For metabolomics sequencing, samples with seven biological replicates of five locusts for both treatment and control groups were prepared at Beijing Biomarker Technologies Co., Ltd (Beijing, China). The metabolite extraction involved the addition of an appropriate volume of extraction solvent along with magnetic beads for grinding and ultrasonic treatment. Centrifugation was performed to collect the supernatant, which was then vacuum‐dried. A suitable volume of extraction solvent was subsequently added for the re‐dissolution of the metabolites prior to analysis. The detection platform used was the Waters Acquity I‐Class PLUS ultra‐high‐performance liquid chromatography coupled with the Waters Xevo G2‐XS QTOF high‐resolution mass spectrometer, with analysis executed according to predetermined parameters. Raw data acquired through MassLynx V4.2 were processed using Progenesis QI software for tasks including peak extraction and peak alignment. Metabolite identification was accomplished using the online METLIN database, public databases, and a self‐constructed library, combined with theoretical fragment recognition for both qualitative and quantitative metabolite analysis. After qualitative and quantitative evaluations of the metabolites, further analyses were performed, including data quality assessment, KEGG annotation analysis, differential expression analysis, and functional enrichment analysis.

### 4.29 RNA Extraction and Quantitative RT‐PCR (qRT‐PCR)

Total RNA was extracted from the flight muscles of adult male locusts using the RNA extraction Kit (Promega (Beijing, China). The RNA integrity was verified by electrophoresis on a 1% (w/v) agarose gel, and the concentration and purity were measured using a Nanodrop 2000 spectrophotometer. Total 1 µg of RNA was reverse‐transcribed into cDNA using the PrimeScript RT Reagent Kit (TaKaRa, China). The relative mRNA expression was assessed with BlasTaq 2X qPCR MasterMix on the Roche LightCycler 480 system following the methods of Huang et al. [105]. A housekeeper gene *RP49* served as the internal reference gene, relative expression levels were analyzed utilizing the 2^−ΔΔCt^ method [11]. All primers are presented in Table S22.

### 4.30 Western Blotting

Total protein was extracted from the flight muscles using an SDS‐Urea reagent. The protein concentration was determined utilizing the PierceTM BCA Protein Assay Kit (Thermo Fisher Scientific, USA) for normalization. The proteins were subjected to 6% SDS‐PAGE and subsequently transferred to a PVDF membrane (Millipore). (Millipore, USA). The membrane was then blocked with a solution of 5% (wt/vol) skim milk powder at room temperature (RT) for 1 h. Subsequently, the membrane was incubated overnight at 4°C with primary antibodies: anti‐P‐AMPKα(T172) (1:5000; CST, 2035 S, USA) and anti‐HSP90 (C45G5) (1:5000; CST, 4877 S, USA). Afterward, a secondary antibody (anti‐rabbit, 1:10 000; CST, 7074 S, USA) was added and incubated with shaking at RT for 1 h. The detection of immunological blotting was conducted using the ECL Western Blot Kit (Thermo Fisher Scientific, USA). The band intensities were the relative gray level when scanned using ImageJ.

### 4.31 Statistical Analysis

Data preprocessing, including normalization and outlier assessment, was performed as described in the corresponding Methods sections. Data are presented as mean ± standard deviation (SD).

Comparisons of nucleotide diversity (π) and Tajima's D between populations were performed using two‐sided Wilcoxon rank‐sum tests. The same test was also used to compare selection statistics, including Z*fst* values and XP‐CLR scores, between candidate adaptive TE insertion regions and genome‐wide regions. Differences in the distribution of TE classes between the reference genome and the candidate adaptive TE set were assessed using chi‐square tests. Comparisons of gene expression between populations and between control and *PIEZO* knockdown groups were performed using two‐sided Student's t‐tests, whereas comparisons of gene expression among developmental stages were performed using one‐way analysis of variance (ANOVA). Differences in flight ability and respiratory rate between groups were evaluated using Mann–Whitney U tests. Comparisons of genomic features across different insect species were performed using one‐way ANOVA followed by Tukey's multiple comparisons test (Figure S3). A significance threshold of *P* < 0.05 was applied unless otherwise specified.

Student's t‐tests and Mann–Whitney U tests were conducted in SPSS (IBM Corp., Armonk, NY, USA). Wilcoxon rank‐sum tests, chi‐square tests, and one‐way ANOVA followed by Tukey's multiple comparisons test were performed in R v4.3.0 [106] using the functions wilcox.test(), chisq.test(), aov(), and TukeyHSD() implemented in the base stats package. Exact sample sizes, *P* values, and statistical tests are reported in the corresponding figure legends or tables.

## Author Contributions

**Bing Chen**: conceptualization; **Xuanzhao Li**, **Ying Liu**, **Longsheng Xing**, **Huilong Du**, and **Bing Chen**: methodology; **Xuanzhao Li**, **Longsheng Xing**, and **Ying Liu**: formal analysis; **Ying Liu**, **Yuze Zhang**, and **Yingming Sun**: investigation; **Huilong Du** and **Bing Chen**: resources; **Xuanzhao Li** and **Lei Yue**: data curation; **Xuanzhao Li**, **Ying Liu**, **Longsheng Xing**, and **Bing Chen**: writing – original draft; **Xianliang Huang**, **Huilong Du**, and **Bing Chen**: writing – review & editing; **Xuanzhao Li**, **Ying Liu**, and **Longsheng Xing**: visualization; **Huilong Du** and **Bing Chen**: supervision; **Bing Chen**: funding acquisition.

## Conflicts of Interest

The authors declare no conflicts of interest.

## Supporting information




**Supporting File 1**: advs76705‐sup‐0001‐SuppMat.docx.

**Supporting File 2**: advs76705‐sup‐0002‐TableS1‐S22.xlsx.

## Data Availability

The data that supports the findings of this study are available in the supplementary material of this article.
