# Supplementary material for: Transposable Element–Driven PIEZO Mutation Enhances Locust Flight in Plateau Hypoxia
Source: Adv Sci (Weinh). 2026 Jul 20:e76705. Online ahead of print. doi: 10.1002/advs.76705 (PMC13383693; doi:10.1002/advs.76705)
Supplement: Supplementary file 1 — Supporting File 1: advs76705‐sup‐0001‐SuppMat.docx. [file ADVS-9999-e76705-s001.docx]

Supporting Information

Transposable Element–Driven *PIEZO* Mutation Enhances Locust Flight in Plateau Hypoxia

Xuanzhao Li, Ying Liu, Longsheng Xing, Xianliang Huang, Yingming Sun, Lei Yue, Yuze Zhang, Huilong Du*, Bing Chen*

Supplementary Material:

Contains Figures S1-S25

Other supporting materials for this manuscript include the following:

Table S1-S22


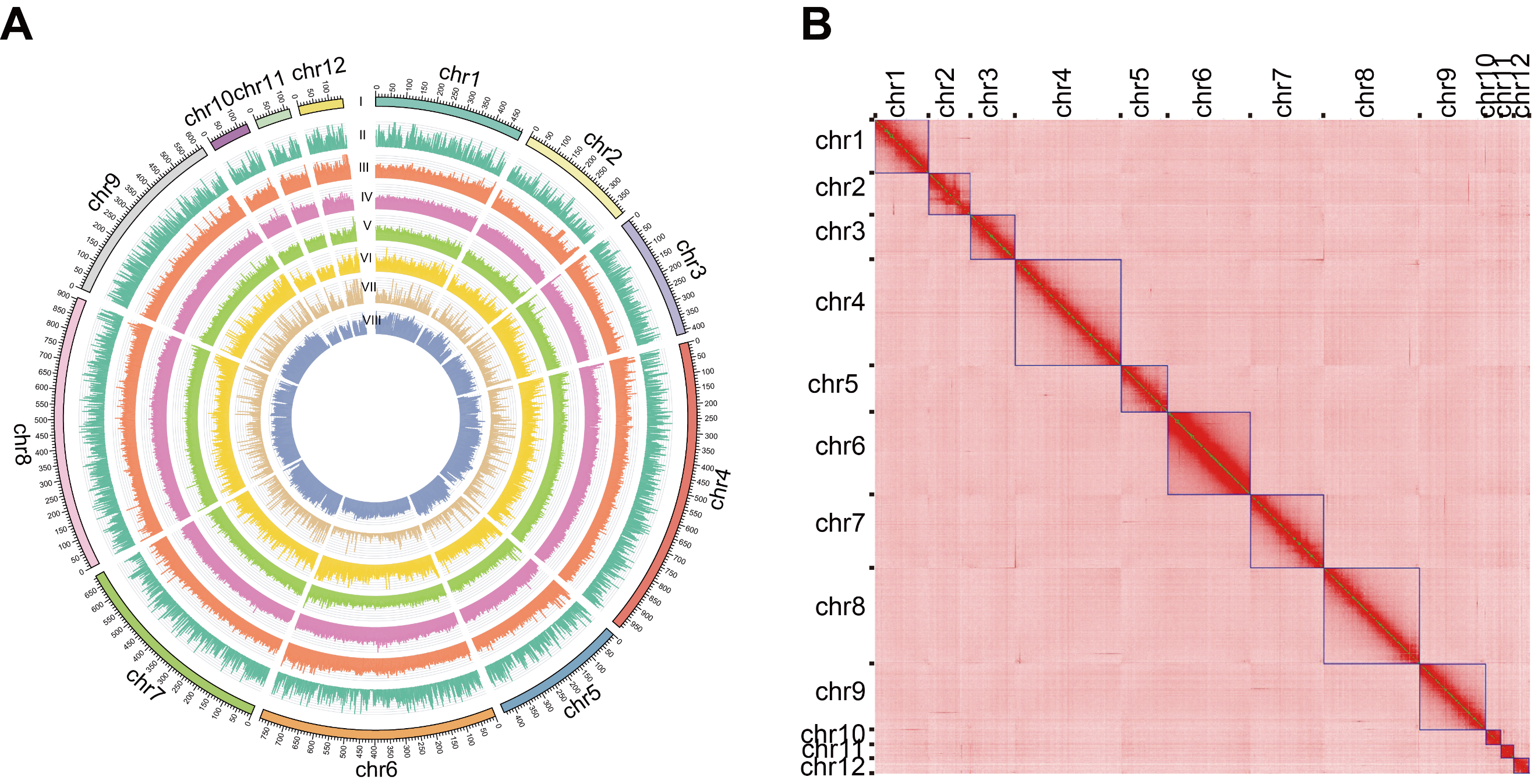


Figure. S1. Overview of the migratory locust genome assembly. (A) Landscape of the genome features of the migratory locust, *Locusta migratoria*, illustrated as a Circos plot. The tracks from the outermost to the innermost represent: (I) chromosome ideogram; (II) protein-coding gene density; (III) G + C content; (IV) DNA transposon density; (V) long interspersed nuclear elements (LINE) density; (VI) long-terminal retrotransposon (LTR)/Gypsy density; (VII) LTR/Copia density; (VIII) genome-wide single nucleotide polymorphism (SNP) density. (B) Chromatin interaction heatmap generated using 3D-DNA and juicer based on Hi-C data.


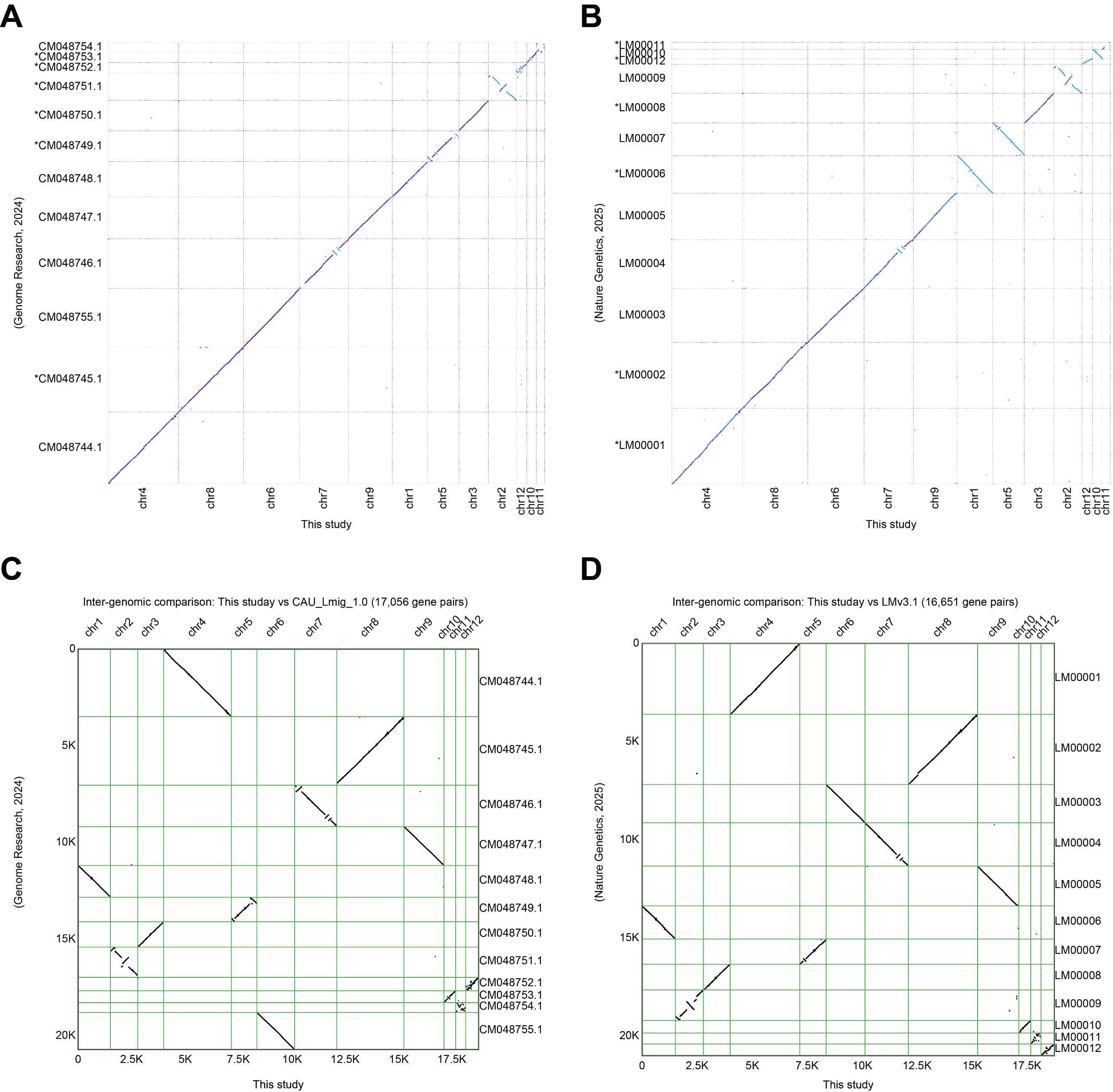


Figure. S2. Genome-wide synteny analysis between our assembly and recently published locust genome. (A-B) Synteny plot based on MUMMER whole-genome alignment. X axis represents the chromosomes of the genome assembly obtained in this study. Y axis represents the chromosomes of the genome assembly obtained in the study published in *Genome Research* (Li et al. 2024) (A) or that in *Nature Genetics* (Liu et al. 2025) (B). (C-D) Genome-wide synteny plot based on JCVI (Python version of MCScan (<https://github.com/tanghaibao/jcvi)>). All genome assemblies were derived from the locust species *Locusta migratoria*.


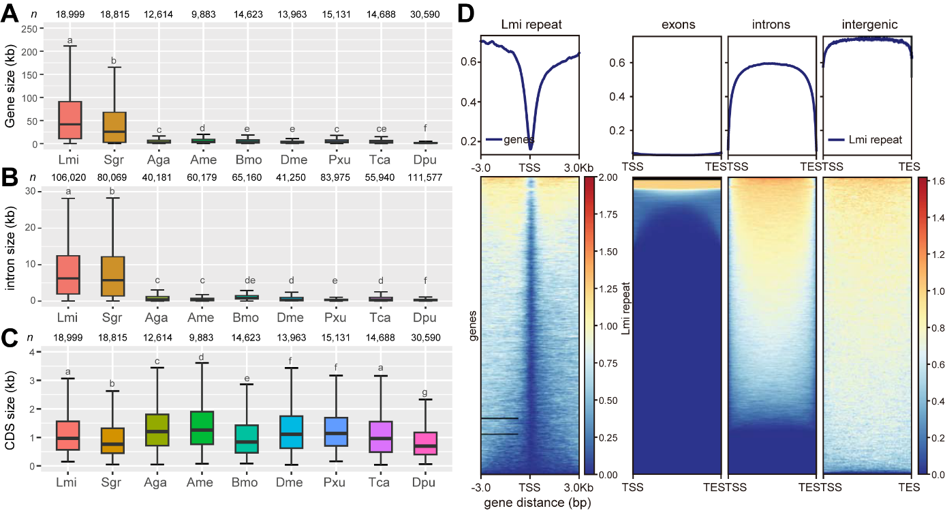


Figure. S3. Comparison of gene features across different insect species and distribution of repeat sequences in different genomic regions of locust genome. (A) Box plot showing the distribution of gene lengths in nine arthropod species. (B) Box plot showing the distribution of intron lengths in nine arthropod species. (C) Box plot showing the distribution of CDS lengths in nine arthropod species. Box plots show the median, interquartile range, and 1.5 × IQR. Sample sizes are indicated above each box plot. Species name abbreviations: Lmi, *Locusta migratoria*; Sgr, *Schistocerca gregaria*; Aga, *Anopheles gambiae*; Ame, *Apis mellifera*; Bmo, *Bombyx mori*; Dme, *Drosophila melanogaster*; Pxu, *Papilio xuthus*; Tca, *Tribolium castaneum*; Dpu, *Daphnia pulex*. Different letters indicate significant differences between examined species. Statistical significance was determined using the Analysis of Variance (ANOVA), followed by Tukey’s multiple comparisons test. (D) Metaplots showing the distribution of repeat sequences 3 kb upstream and downstream of transcription start site (TSS) and other genomic regions.

~~
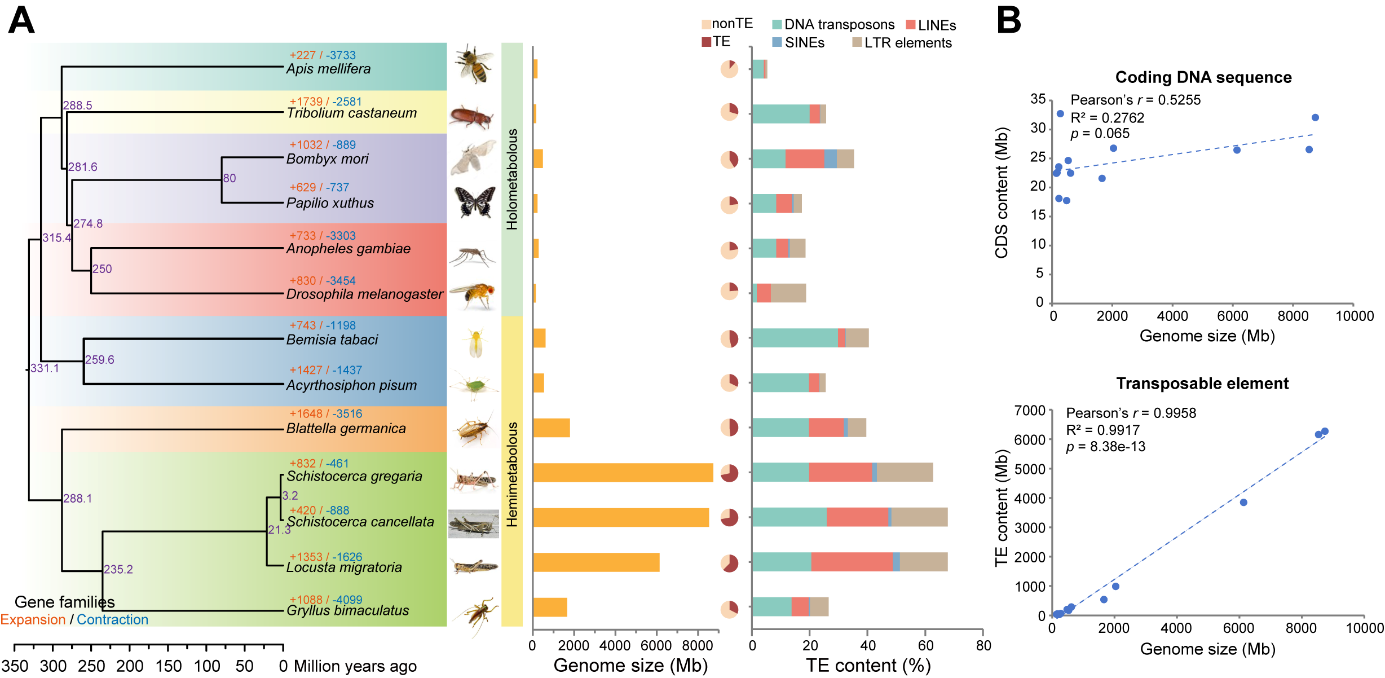
~~

Figure. S4. Distribution of different types of repeat sequences in *L. migratoria* and twelve other insect species. (A) Phylogenomic analysis of the migratory locust and twelve other insect species. The phylogeny was constructed based on the strict single-copy orthogroups that were obtained from six holometabolous and seven hemimetabolous insect species. Gene family evolution analysis was conducted to identify the gain and loss of gene families across distinct lineages using CAFÉ 4. The numbers in the inner nodes indicate the calibrated species divergence time in a time unit of million years. Genome size of each species and fraction of TEs (brown) in the middle panel. The distribution of different TE types was displayed in the right panel. (B) The contribution of TE and CDS to genome size variation across insect species, respectively.


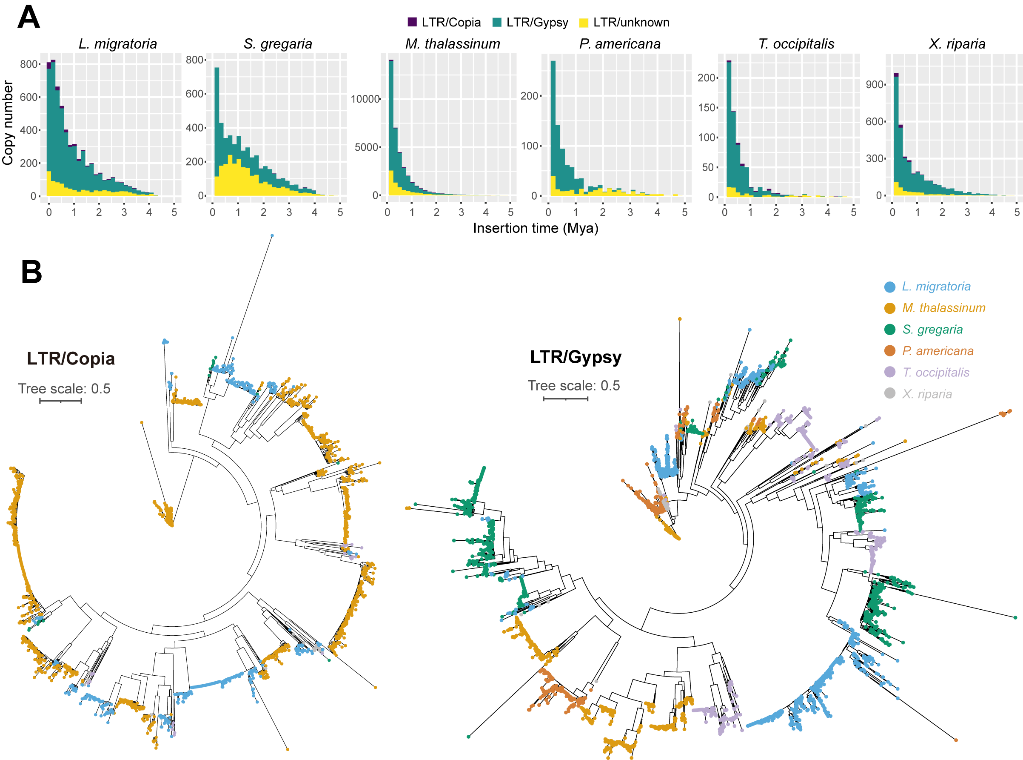


Figure. S5. Composition and evolution of long terminal repeat retrotransposons (LTR-RTs) in locust and closely related species. (A) Stacked bar plots showing the distribution of copy numbers of intact LTR-RTs in six insect species. (B) Maximum likelihood phylogeny of intact LTR/Copia and LTR/Gypsy in six insect species using FastTree.


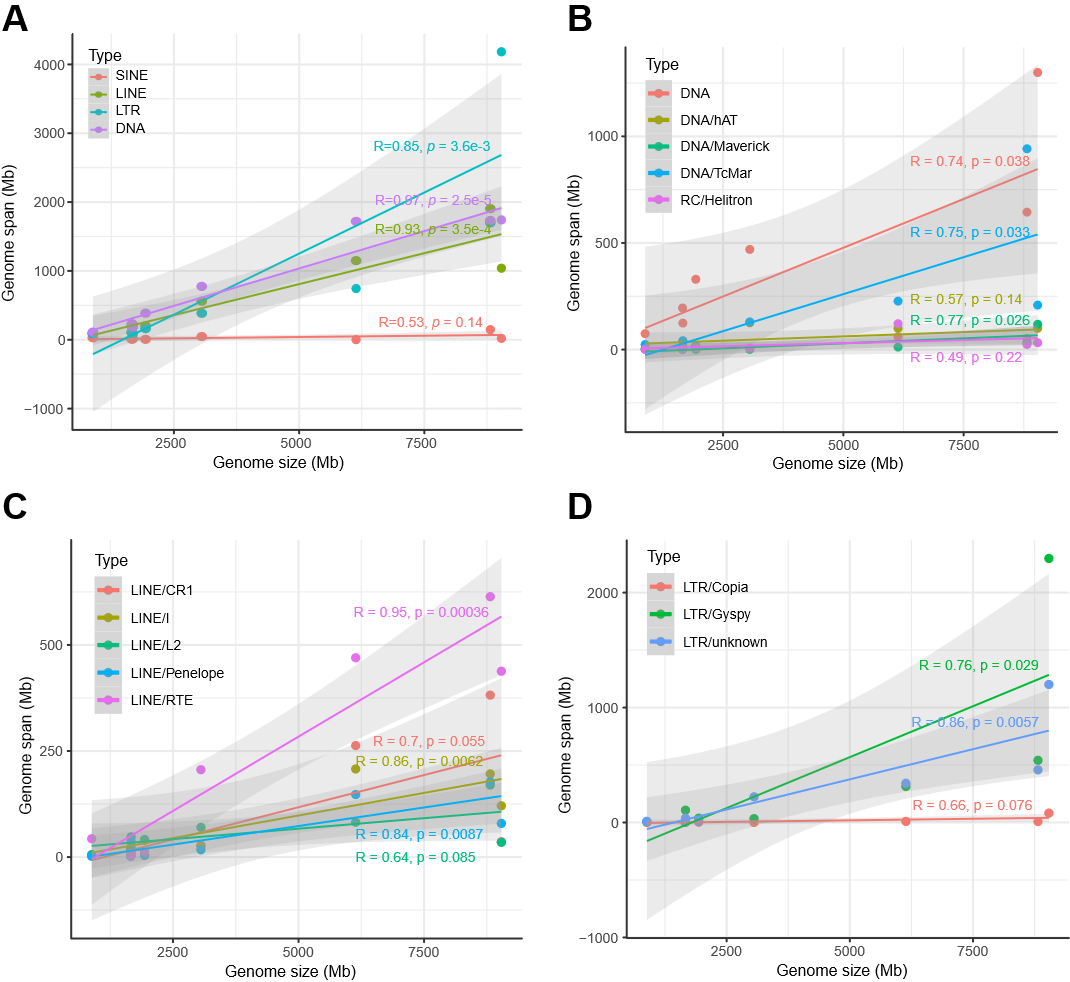


Figure. S6. Correlation between genome size and various types of transposable elements (TEs) in different classification levels. (A) Correlation analysis between genome size and four orders of TEs, including DNA transposons, long interspersed nuclear elements (LINEs), short interspersed nuclear elements (SINEs), and long terminal repeat retrotransposons (LTR-RTs). (B) Correlation analysis between genome size and five superfamilies of DNA transposons. (C) Correlation analysis between genome size and five superfamilies of LINEs. (D) Correlation analysis between genome size and three superfamilies of LTR-RTs.


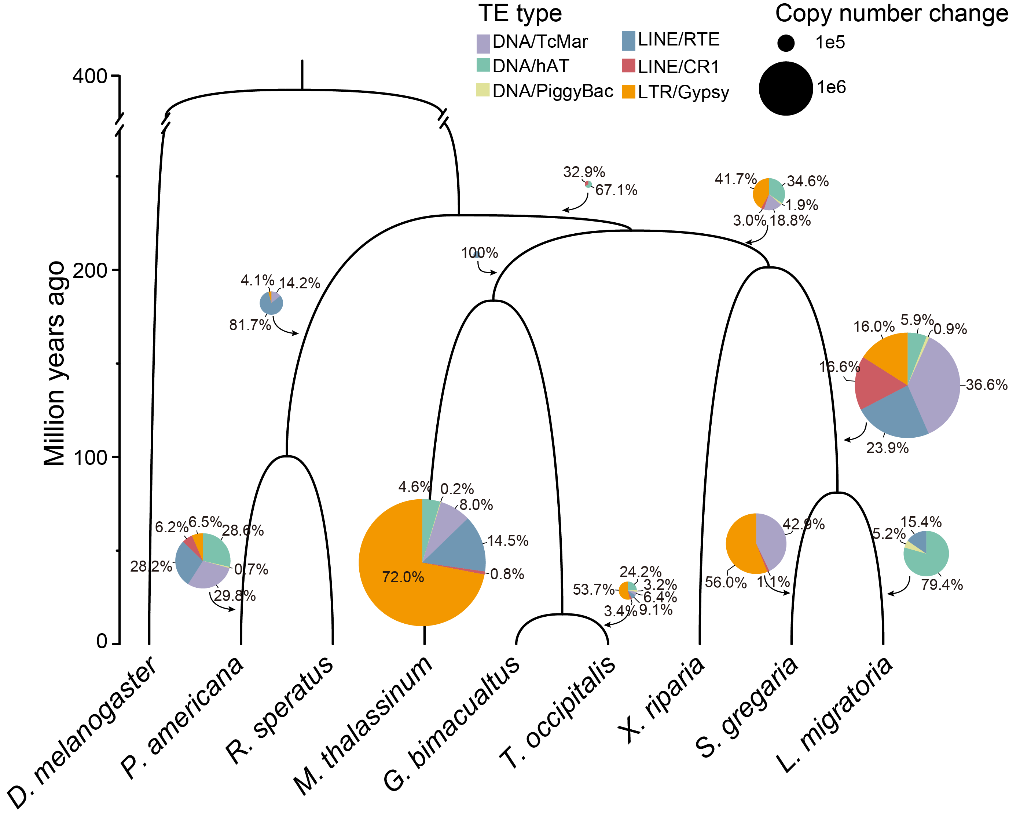


**Figure. S7.** **Schematic representation of the copy number change of six TE superfamilies in the phylogenetic context.** The analysis is based on a proposed model that TE copy sustainedly increased over the evolutionary process. The size of pie chart is proportional to the copy number change of each TE superfamily.


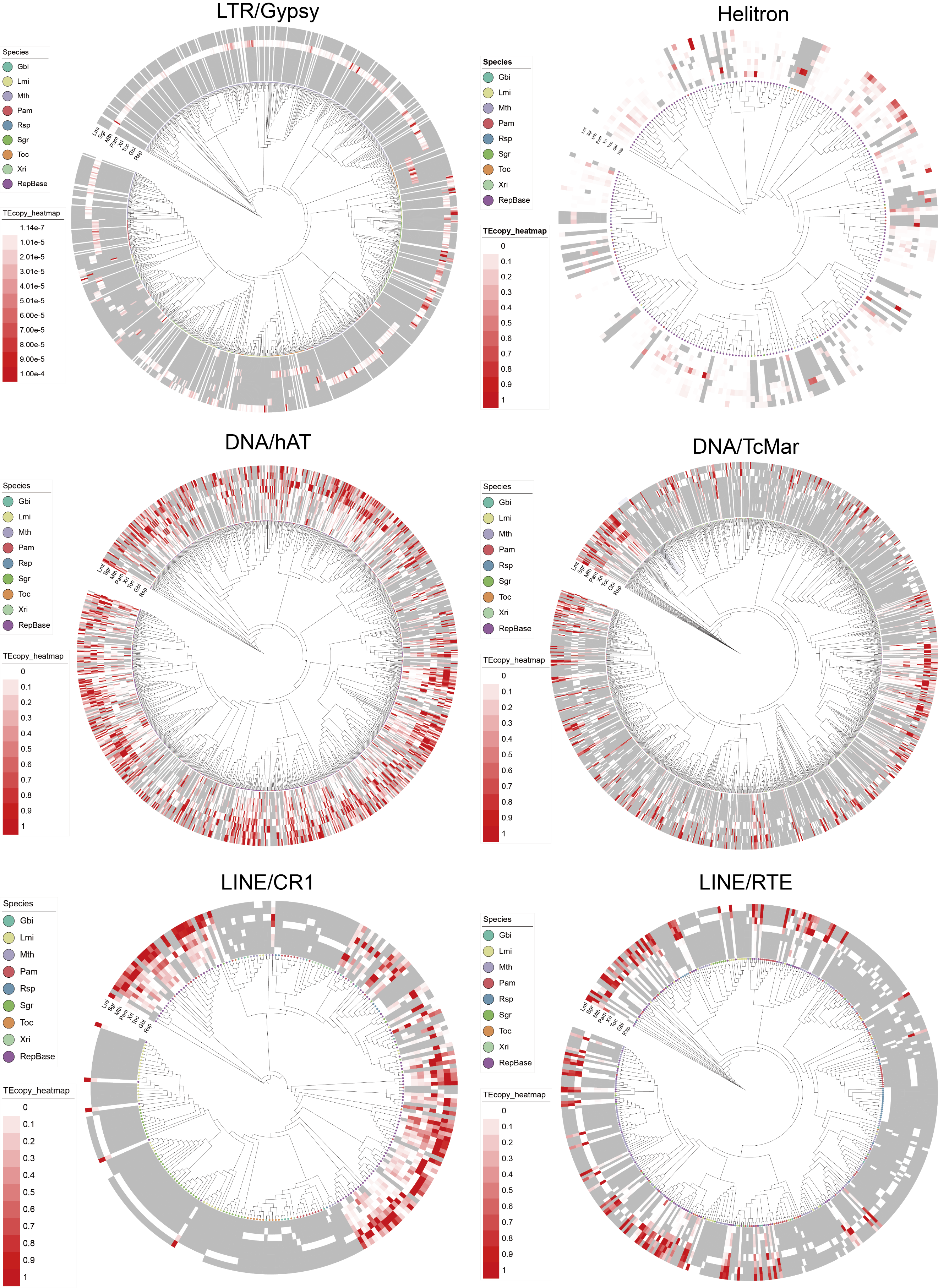


Figure. S8. Phylogenetic tree of common and species-specific TE superfamily consensus sequences. Helitron, DNA/hAT, DNA/TcMar, LINE/CR1, and LINE/RTE were constructed using TE consensus sequences, LTR/Gypsy was constructed using RT domain sequences extracted from a non-redundant full-length LTR library (LTRlib). Gray indicates a value of zero, whereas white denotes very small but non-zero values.


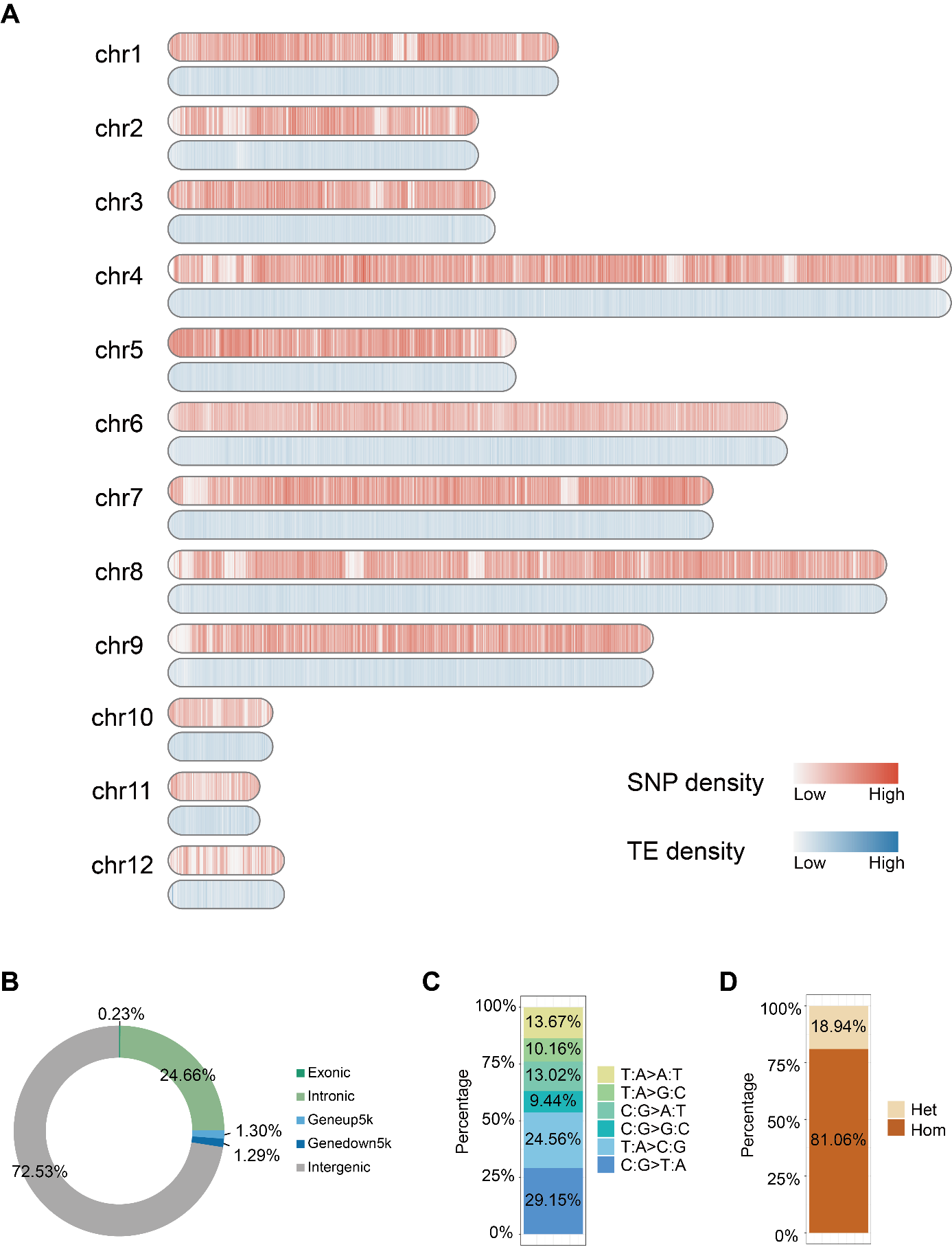


Figure. S9. SNP variant information statistics. (A) The number of SNPs and TEs within 100kb window size. (B) Percentage of SNPs number in different genomic regions. (C) Percentage of different types of nucleotide mutations. (D) Percentage of homozygous and heterozygous single-nucleotide polymorphism (SNP) mutations. Hom, homozygous; Het, heterozygous.


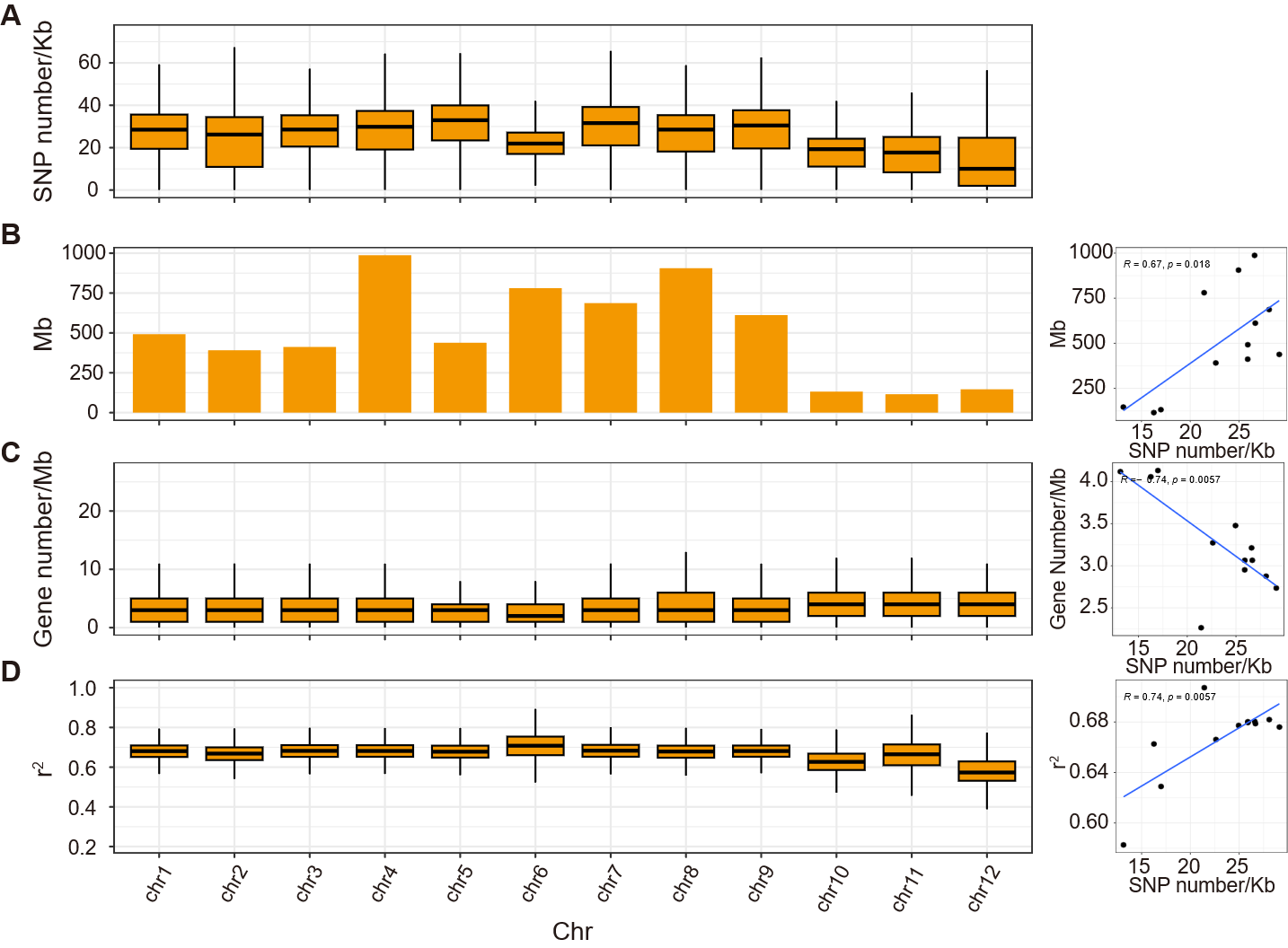


Figure. S10. Chromosome-level variation in genome architecture and population genetic parameters in the migratory locust. (A) SNP density across chromosomes, measured as variants per kb. (B) Chromosome length and its relation with SNP density. Left, DNA length of the 12 chromosomes (Mb); right, Pearson correlation between SNP density and chromosome length. (C) Gene density and its relation with SNP density. Left, gene density across chromosomes, measured as gene number per Mb; right, Pearson correlation between SNP density and gene density. (D) Linkage disequilibrium (LD) and its relation with SNP density. Left, LD distributions across chromosomes, represented by r² values; right, Pearson correlation between SNP density and r^2^. Pearson’s r and corresponding *p* values are shown in each correlation plot.


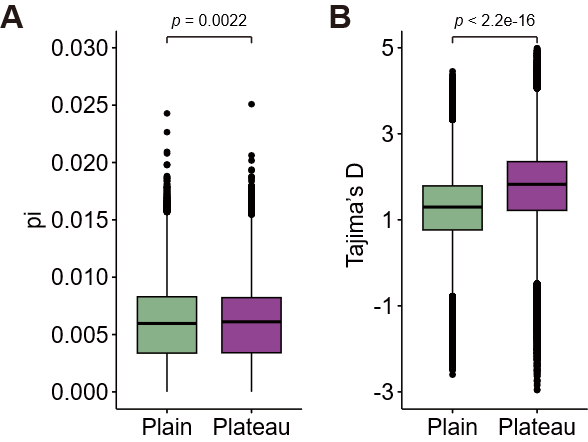


Figure. S11. Comparison of nucleotide diversity (π) and Tajima’s D between subgroups. (A) Significant difference in different population nucleotide diversity (π), as evaluated using the Wilcoxon rank-sum test (*p* = 0.0022). (B) Significant difference in different population Tajima’s D, as evaluated using the Wilcoxon rank-sum test (*p* < 2.2 × 10^-16^). Data plotted using 100kb windows and 50 kb sliding step (n = 122,954 windows).


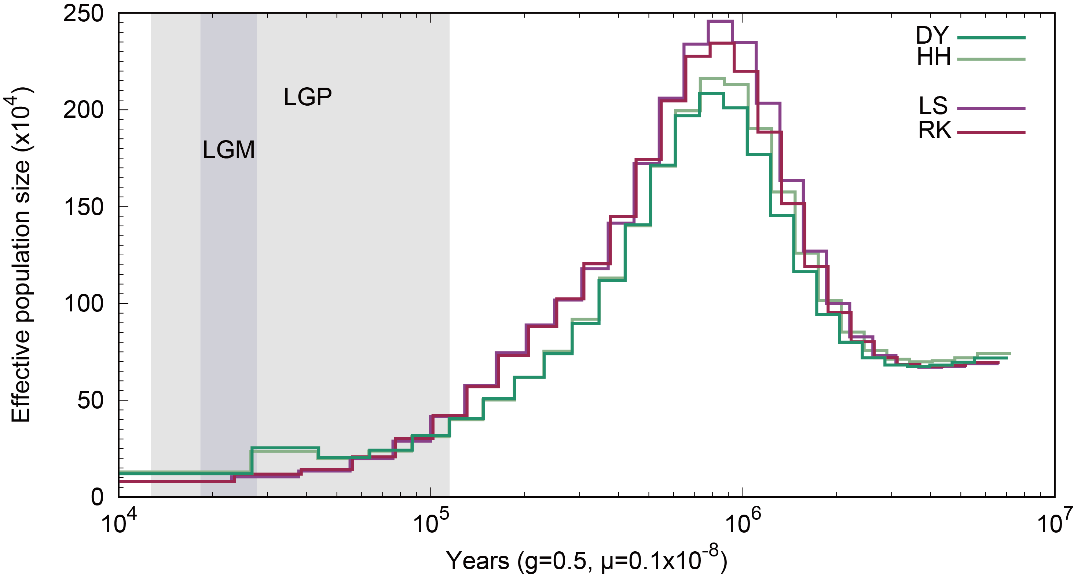


Figure. S12. Demographic history analysis for the four locust populations (HH, DY, LS, and RK). Effective population size of the four populations was estimated using a PSMC model. The x-axis represents years before present on a log scale. LGM represents the last glacial maximum, LGP represents the last glacial period.


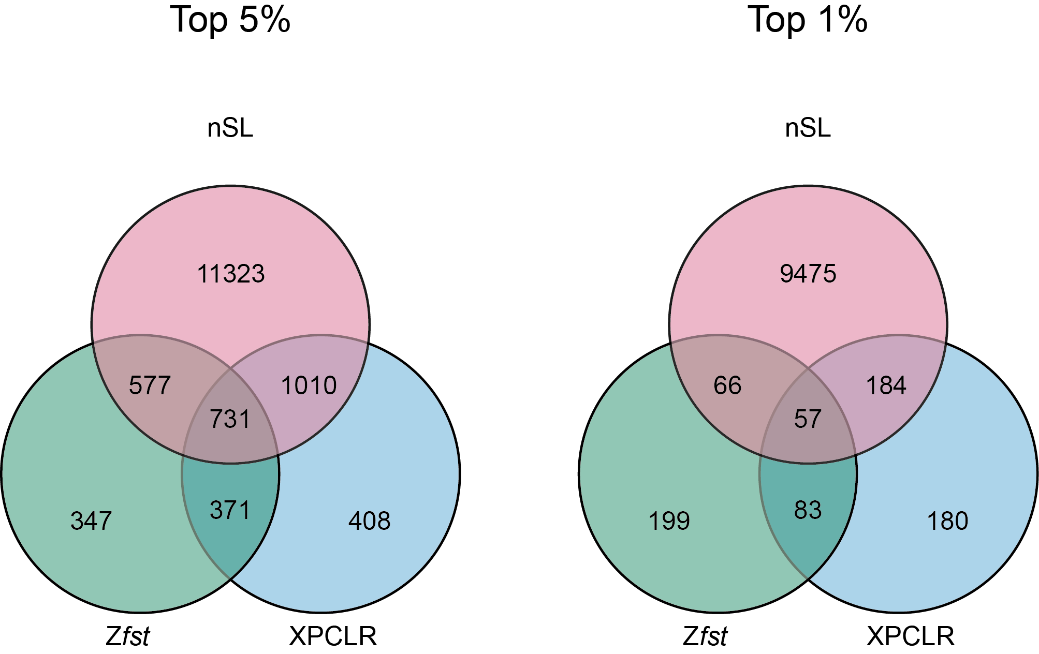


Figure. S13. Venn plot of candidate genes identified by Z*fst*, XP-CLR, and nSL analyses. Candidate genes identified by Z*fst* and XP-CLR were based on genomic windows of 100 kb with a 50 kb sliding step, and genes located within the top-ranking windows were retained. For nSL, scores were calculated for individual SNPs, and genes containing at least one SNP within the top-ranking nSL signals were defined as candidate genes. The left panel shows overlaps among candidate genes identified using the top 5% threshold, whereas the right panel shows overlaps based on the top 1% threshold.


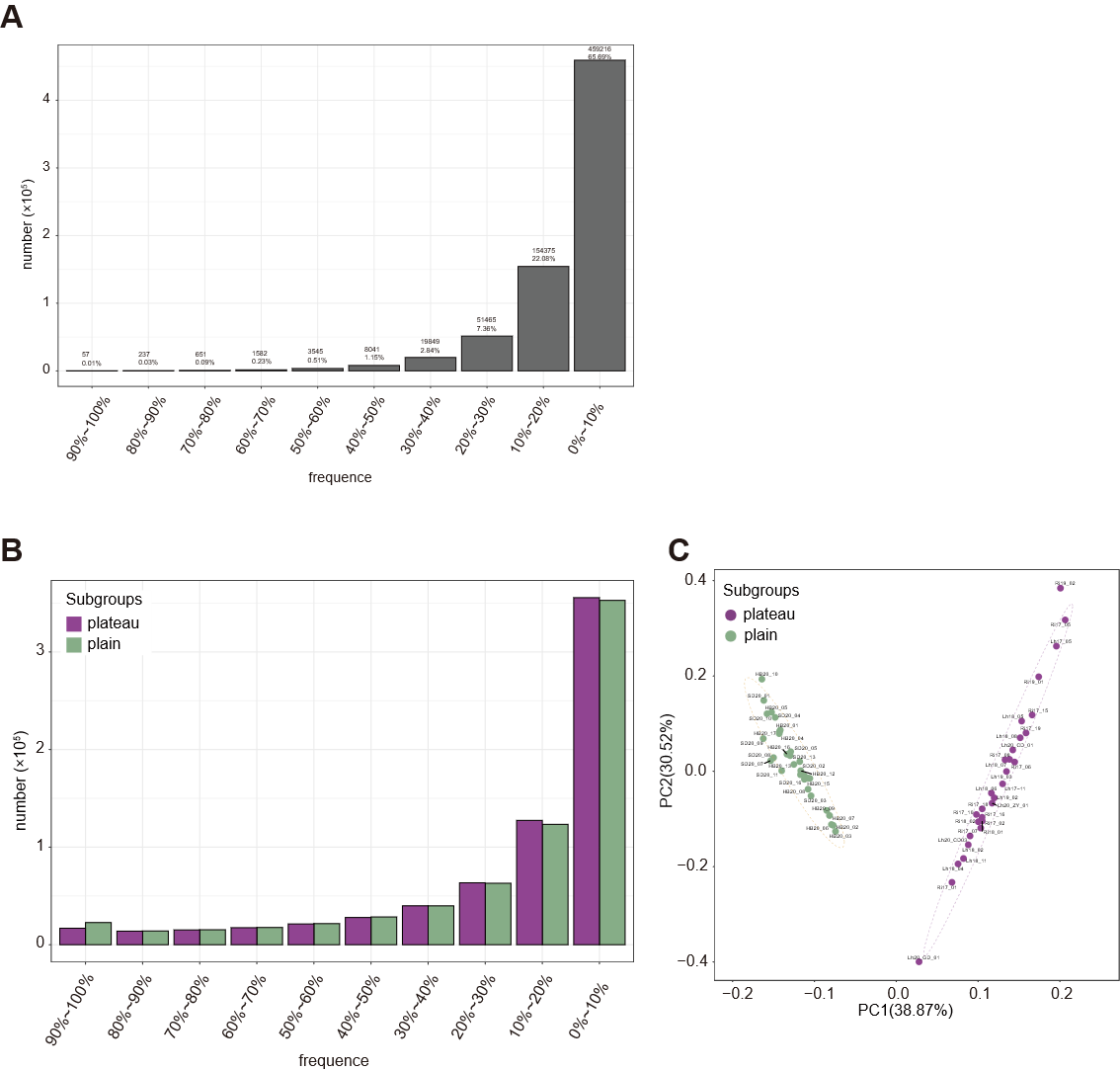


Figure. S14. Frequency distribution statistics of TIPs and PCA based on TIPs. (A) Number and percentage of TE frequency differences. (B) Number and percentage of TE for different population. (C) PCA based on TE insertion polymorphisms (TIPs).


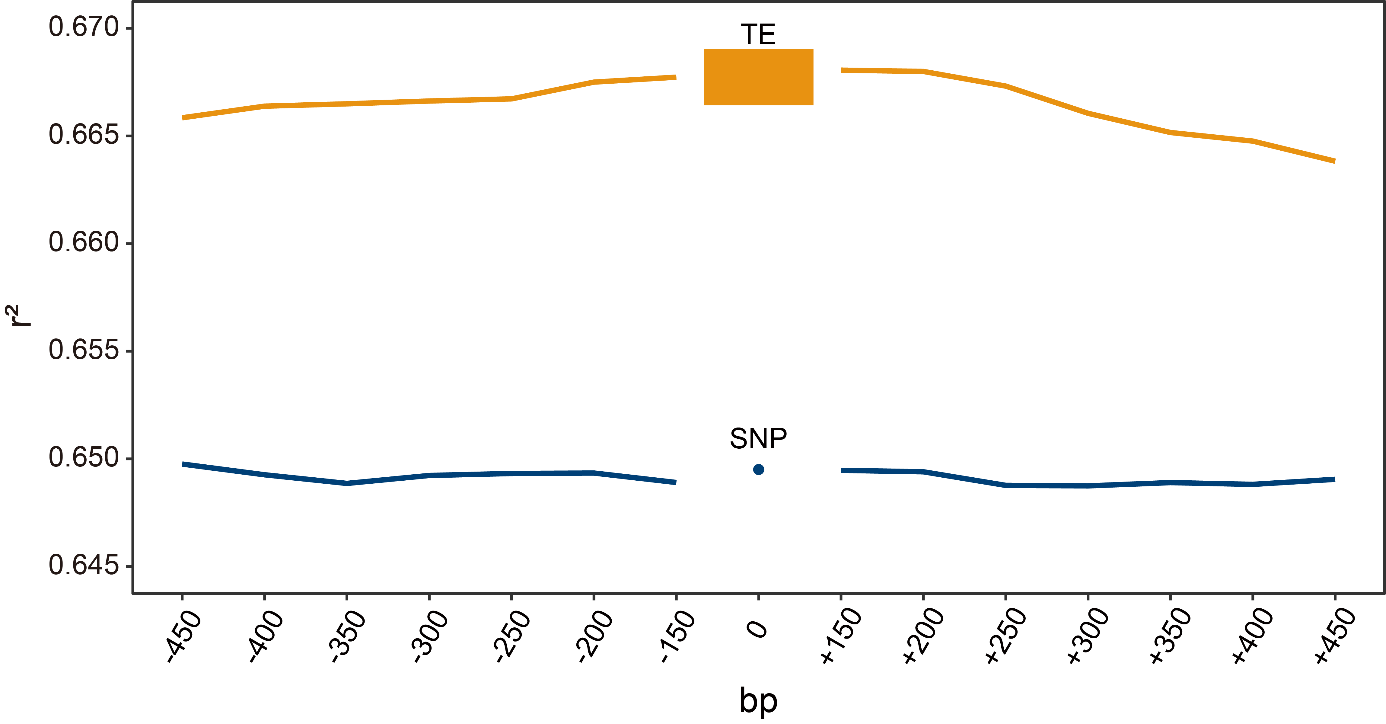


Figure. S15. Local linkage disequilibrium (LD) patterns surrounding candidate adaptive TE insertions and associated SNPs. The plot shows the average r² values across genomic regions flanking candidate adaptive TE insertions (orange line) and nearby SNPs exhibiting strong signatures of selection (blue line). The orange box indicates the TE insertion region, and the blue dot indicates the focal SNP position. The x-axis represents the distance from the TE insertion site or focal SNP. LD surrounding candidate adaptive TE insertions gradually decreases with increasing distance from the insertion site, whereas LD surrounding the associated SNPs remains relatively constant across the analyzed interval. These contrasting patterns are consistent with candidate adaptive TE insertions being located closer to the center of the selected region than the associated SNPs.


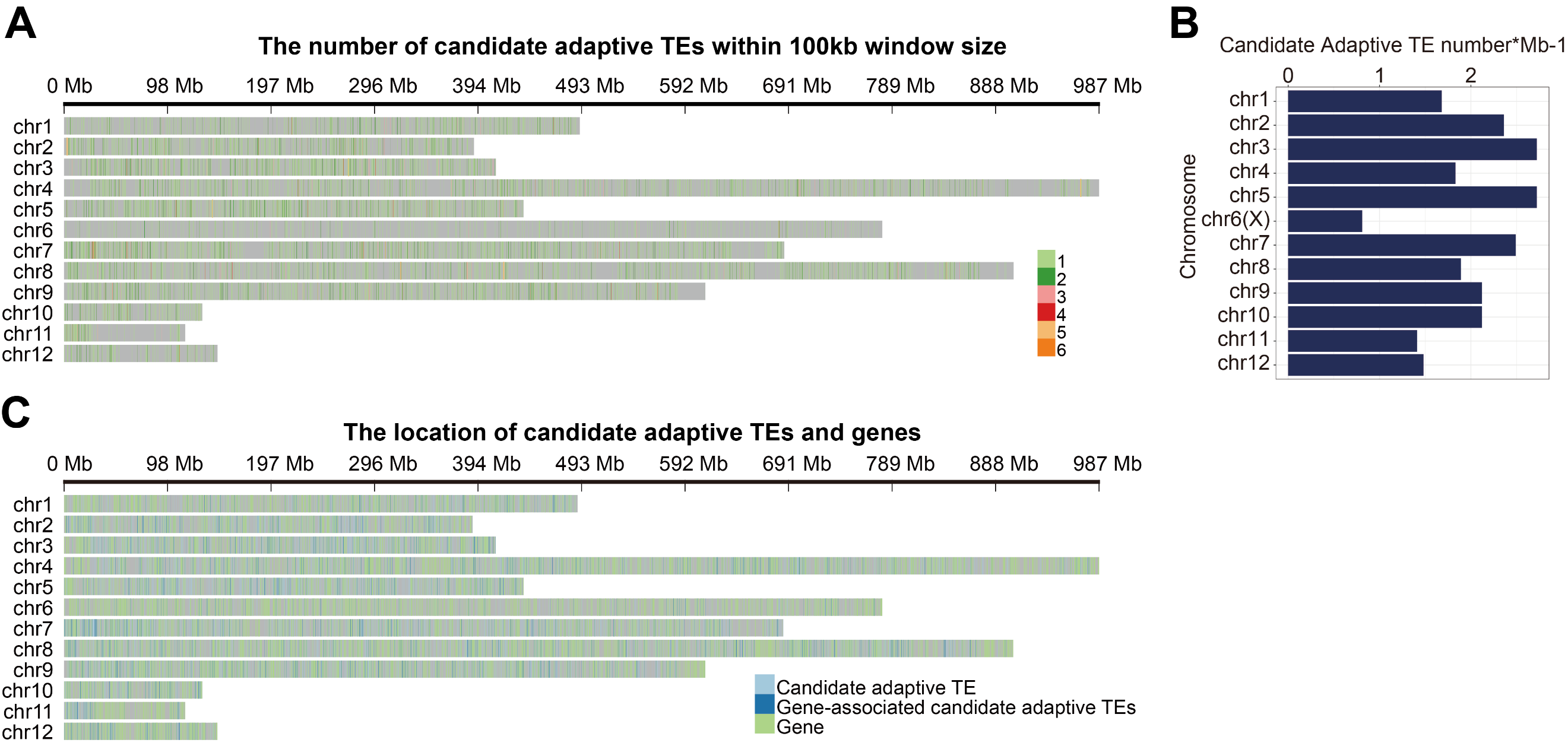


Figure. S16. Adaptive TE information statistics. (A) The number of candidate adaptive TEs within 100kb window size. (B) Candidate adaptive TEs density on each chromosome. (C) The location of candidate adaptive TEs and genes.


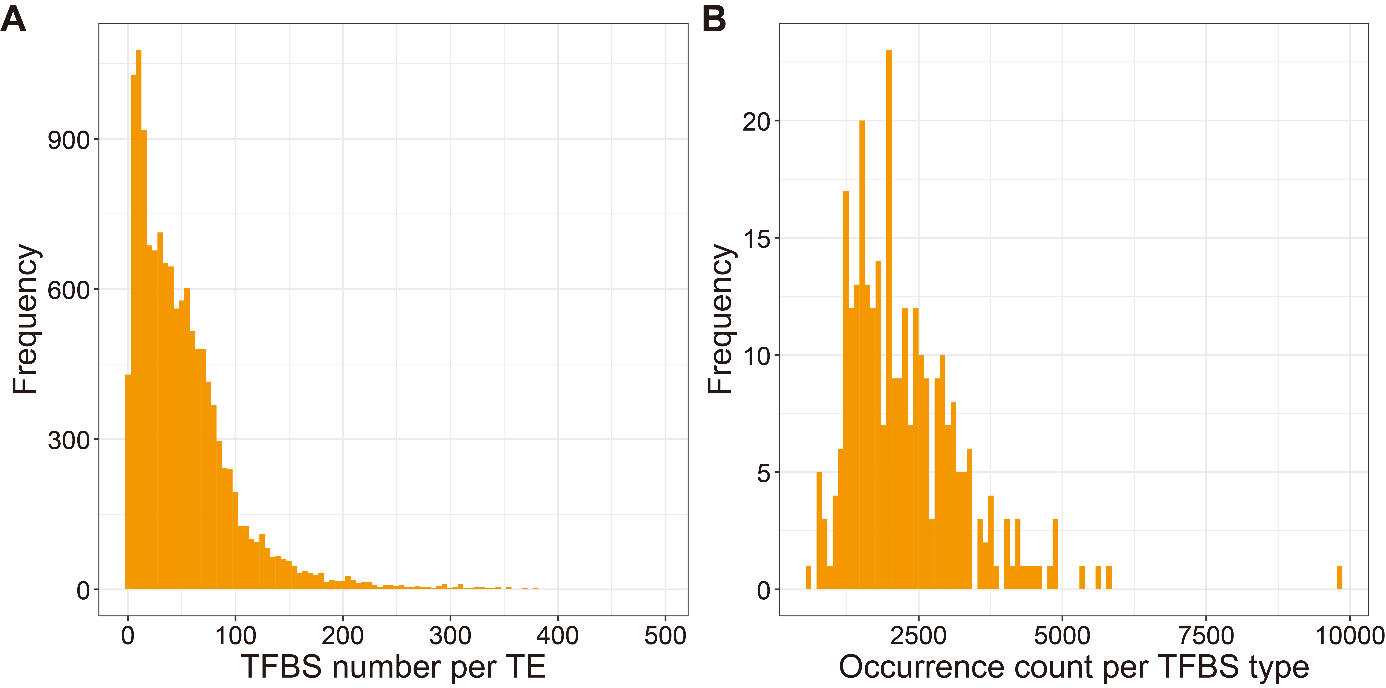


Figure. S17. Statistics of transcription factor binding sites within candidate adaptive TEs. (A) Frequency distribution of transcription factor binding sites per TE. (B) Frequency distribution of occurrences of different transcription factor binding sites.


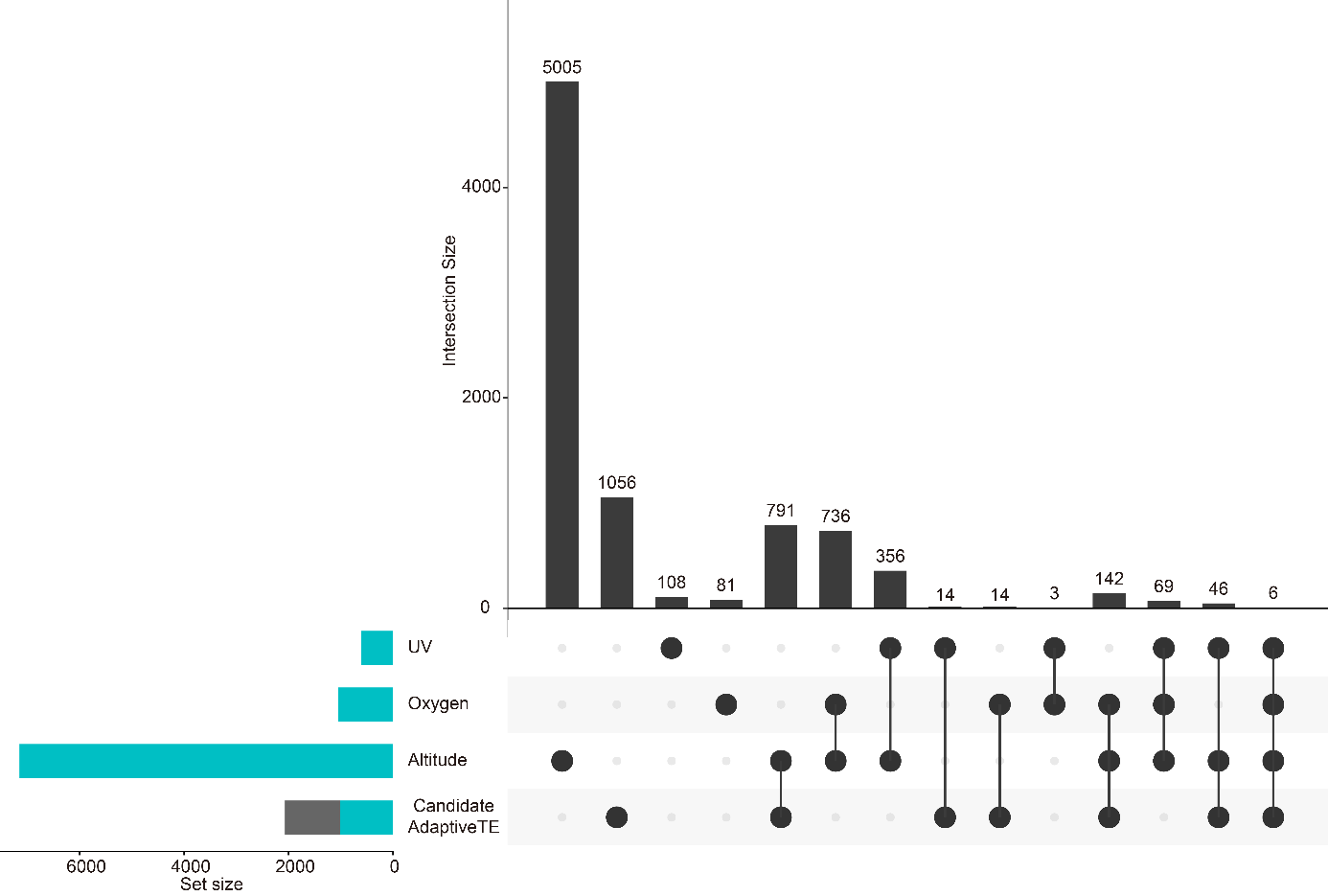


Figure. S18. Upset plots of differentially expressed genes and genes affected by candidate adaptive TE. Altitude: genes differing in basal expression in one tissue of male locusts from plateau and plain populations. Locusts derived from the two populations were reared under the same standard condition (See M&M). On the 3rd day since emergence, four tissues (i.e., brain, flight muscle, ovary and midgut) of male adults was dissected for RNA preparation and transcriptome sequencing. UV: genes differing in expression in cuticles of locusts reared under UV exposure and normal light. Three-d-old adults were subjected to UV stress by exposing to 260 μW/cm² radiation for 14 h daily. Oxygen: genes differing in expression in flight muscle of locusts subjected to normoxic and hypoxic air treatment. Five-d-old adults were subjected to hypoxia stress by exposing to 10 kPa oxygen partial pressure for 48 hours. Genes whose expression differ between treatments with |Fold change| >1 and p value < 0.05 are considered DEGs.


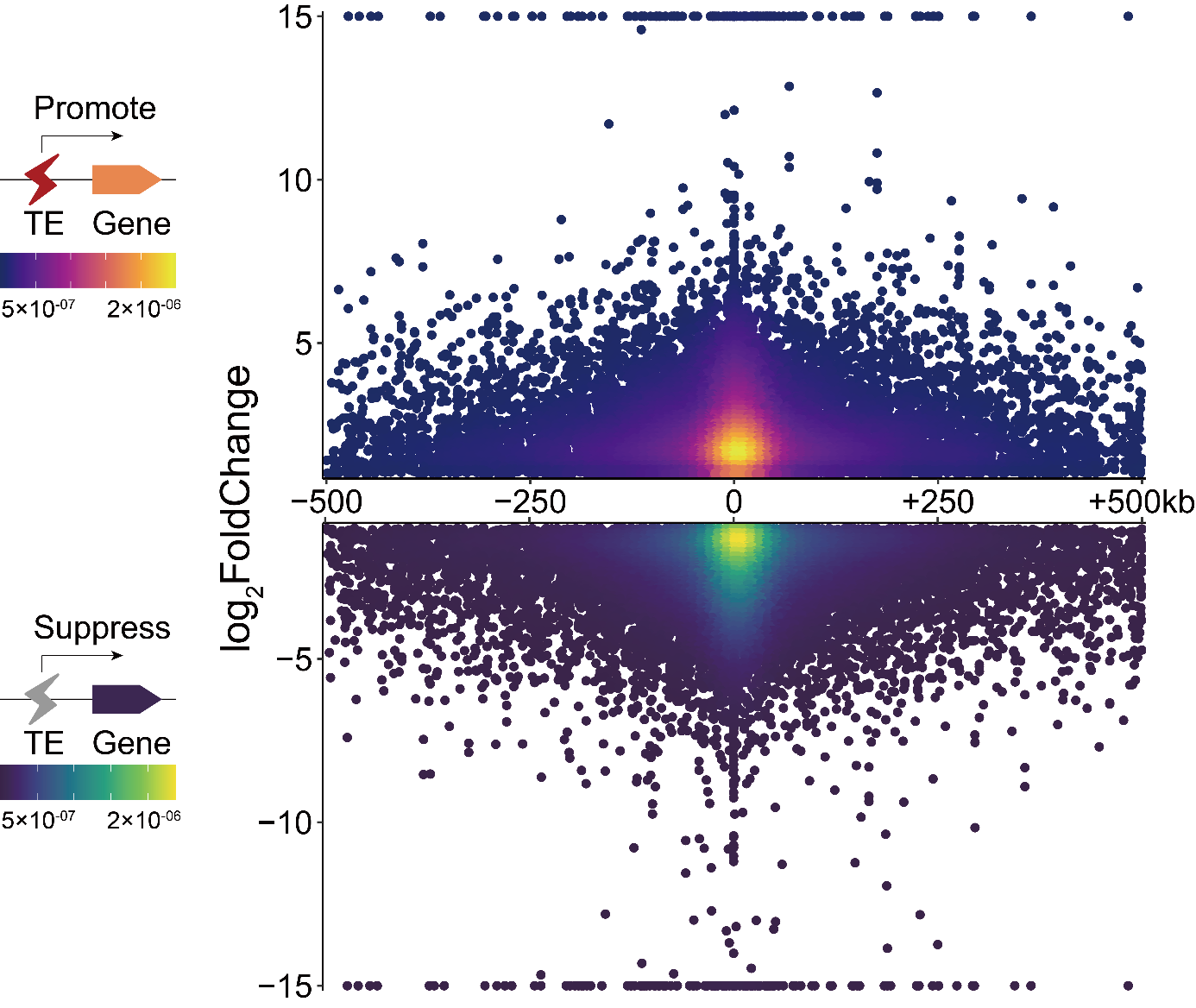


Figure. S19. Expression of candidate adaptive TE-influenced genes in plain and plateau populations. Each point represents one differentially expressed gene. The x-axis indicates the distance between the gene and the candidate adaptive TE, with a value of 0 indicating that the candidate adaptive TE is located within the gene. The y-axis shows the log_2_FoldChange of gene expression in plateau populations relative to plain populations. The color gradient represents the local density of gene points, equal to the number of genes per se.


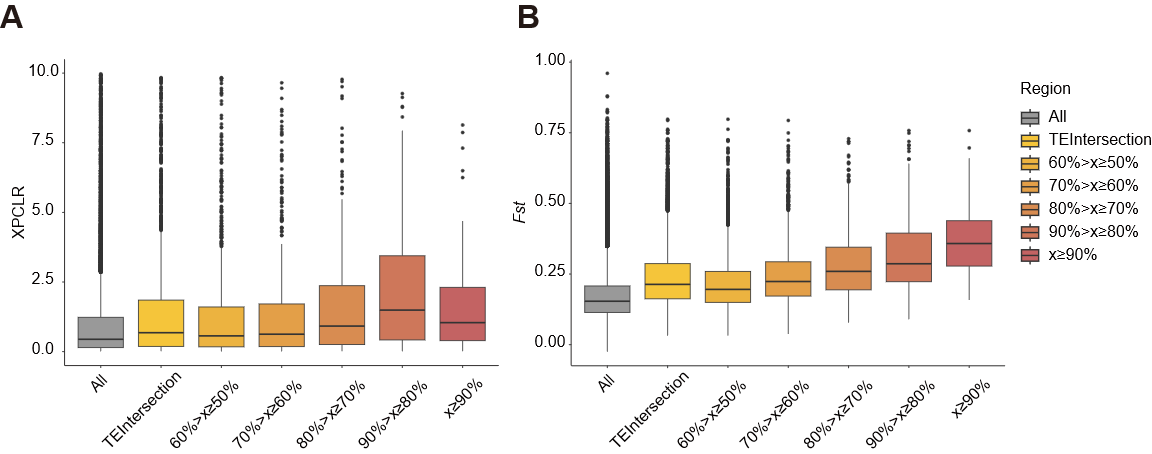


Figure. S20. Statistics of different categories of TEs and relationship between frequency of TE insertion and intensity of selection. Based on their insertion frequency difference, we subdivided these TEs into five categories: 60% > x ≥ 50%, 70% > x ≥ 60%, 80% > x ≥ 70%, 90% > x ≥ 80%, and x ≥ 90%, where x denotes the insertion-frequency difference between the Plateau and Plain groups. We then examined the selection statistic of the DNA region where these TE insertions occurred. (A) Selection evaluation using XP-CLR score in the different adaptive TE insertion regions and the genome-wide region. (B) Selection evaluation using *Fst* value in the different adaptive TE insertion regions and the genome-wide region.


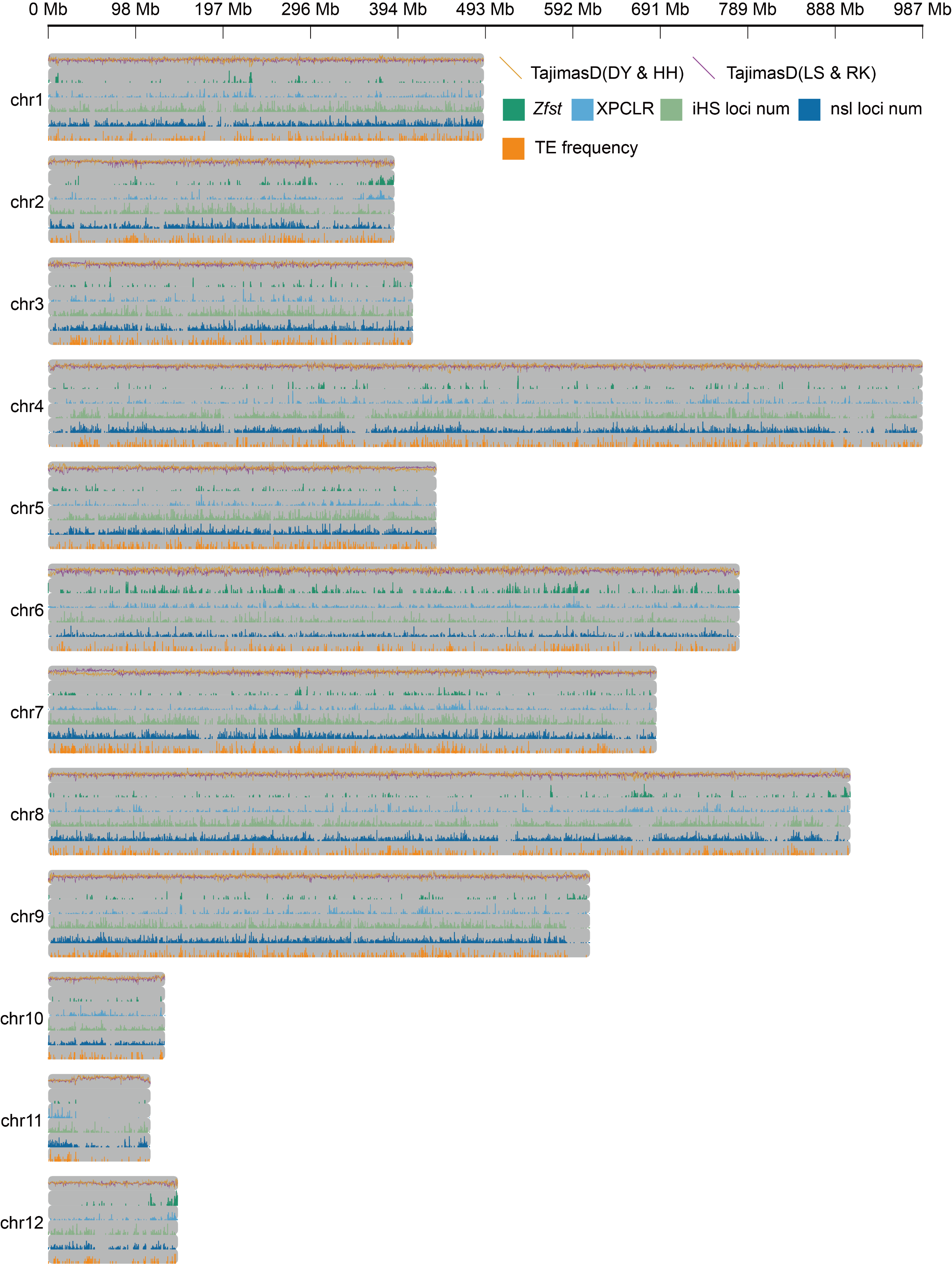


Figure. S21. The landscape of genomic selection. Tajima’s D, Z*fst*, and XPCLR plotted using 100kb windows and 50 kb slide. The iHS and nSL number represents the number of significant sites (value > P95%) screened by the iHS or nSL method within a 100kb window. Orange lines represent the difference between different population adaptive TEs.


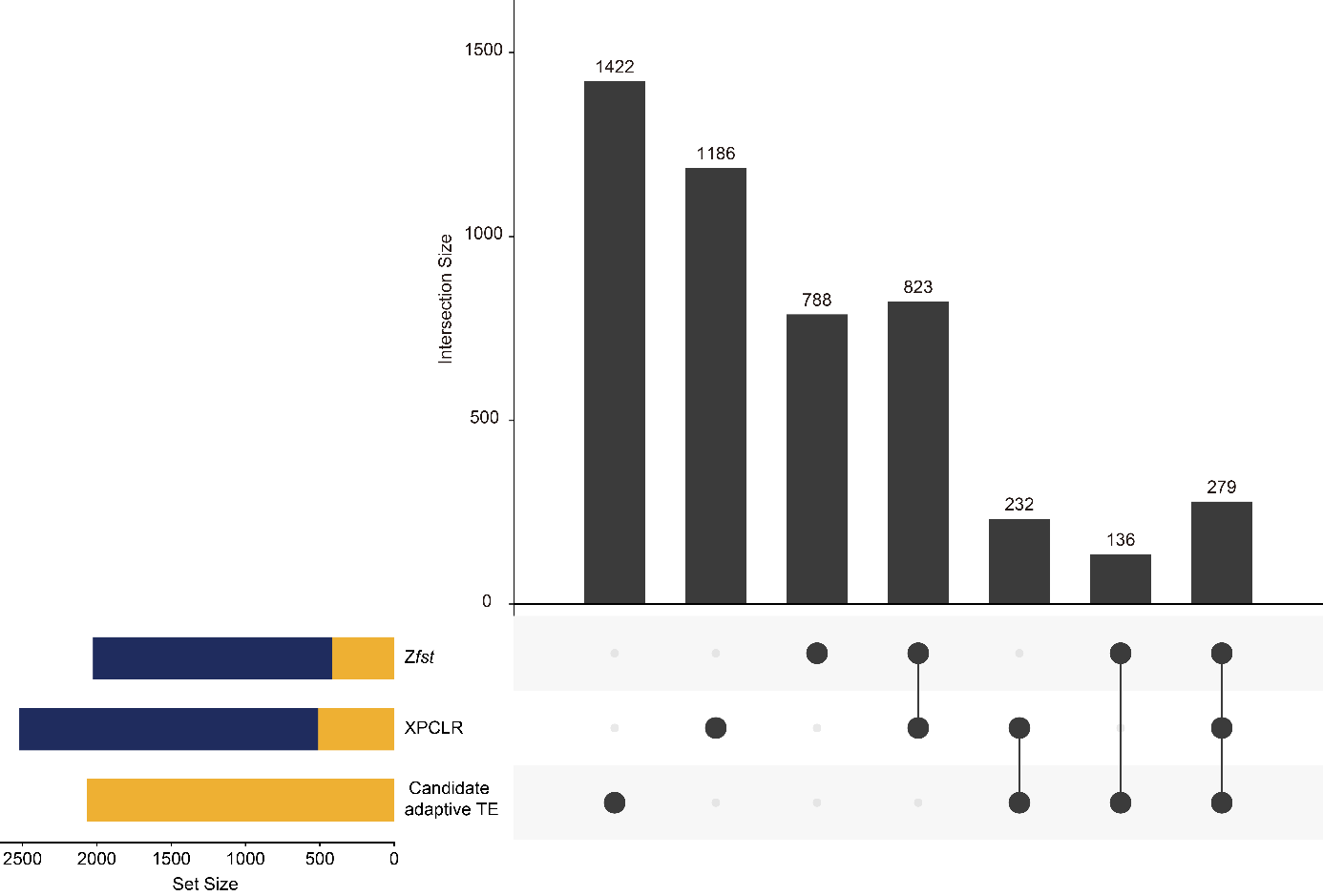


Figure. S22. Upset plots of candidate genes under selective sweeps identified by the three methods.


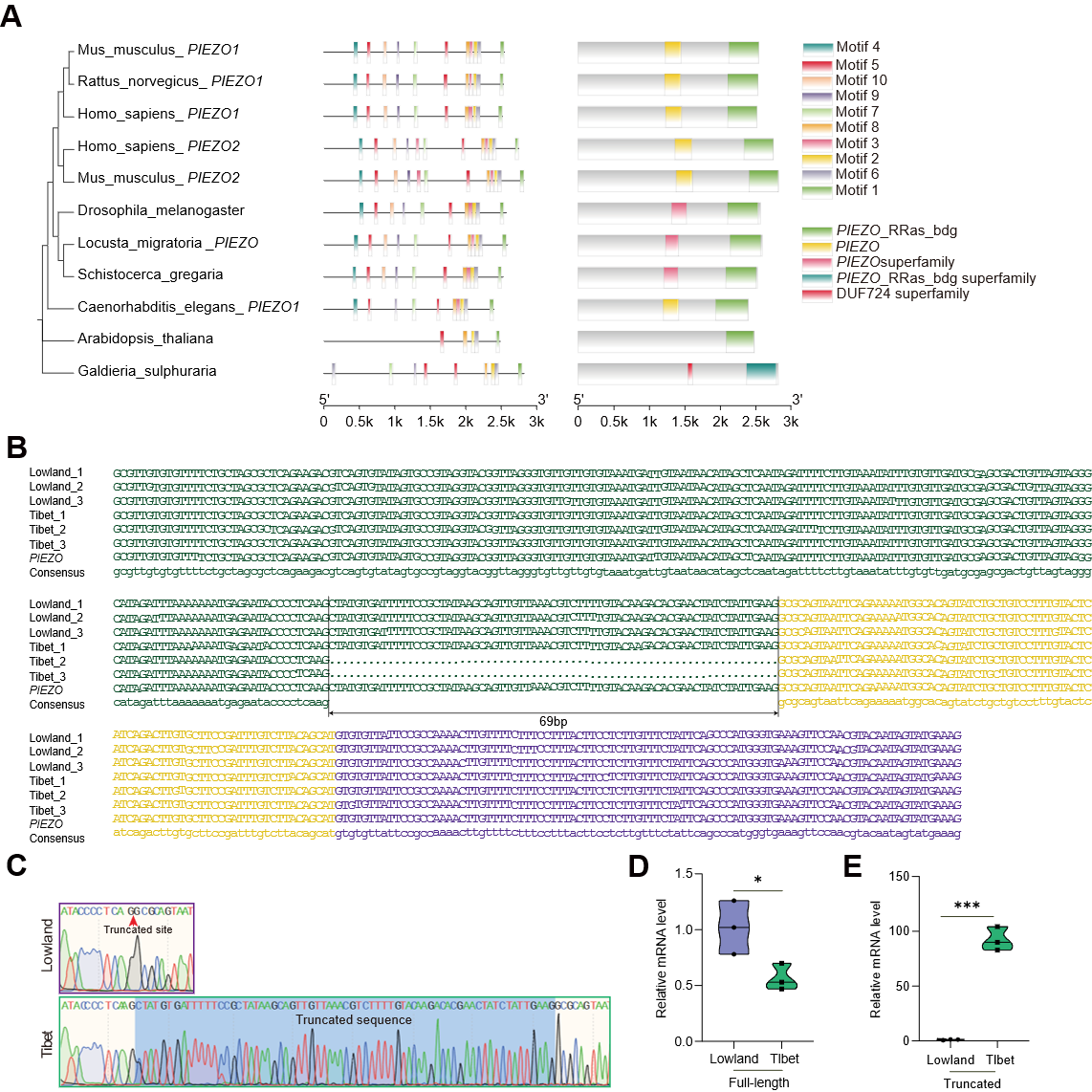


Figure. S23. Evolutionary and alternative splicing analysis of the *PIEZO*. (A) Phylogenetic tree of different species of *PIEZO*. One thousand bootstraps were performed. Only bootstrap values > 85 is shown. (B) Sequence alignment of transcripts at different elevations. (C) Validation of alternative splicing using genomic DNA PCR amplification and Sanger sequencing. Up: Display of truncated loci in Tibet population (The red arrow indicates the sanger sequencing map of the truncated start site at Tibet). Down: Truncated sequence display of Tibet population (The blue areas represent the truncated sequence generated by the PiggyBac insertion in Tibet). (D-E) Expression of full-length and truncated transcripts transcripts of *PIEZO* in lowland and Tibet single-headed locusts. (The values are mean ± SD, **p* < 0.05, ****p* < 0.001 by Student’ s t test. n=3 replicates, one locust per replicate).


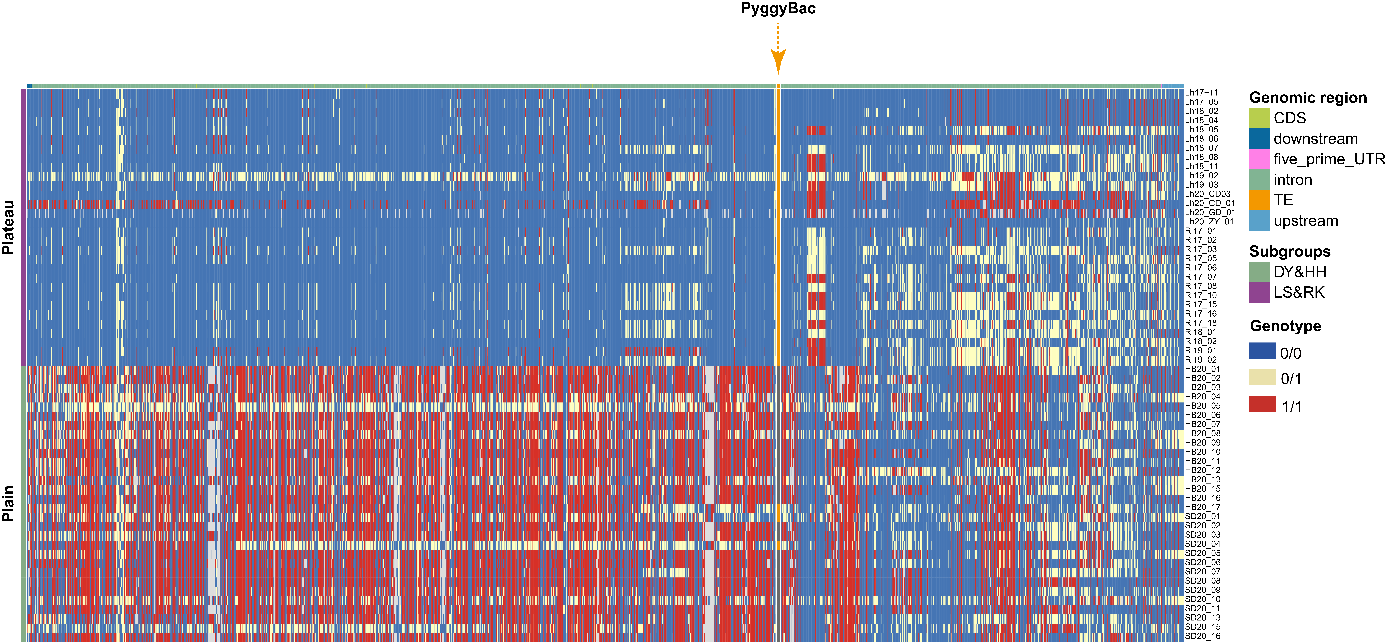


Figure. S24. Genotype heatmap of the *PIEZO* gene region and the 5-kb regions upstream and downstream. The upper most line shows the gene region of *PIEZO*, with annotation of gene structure.


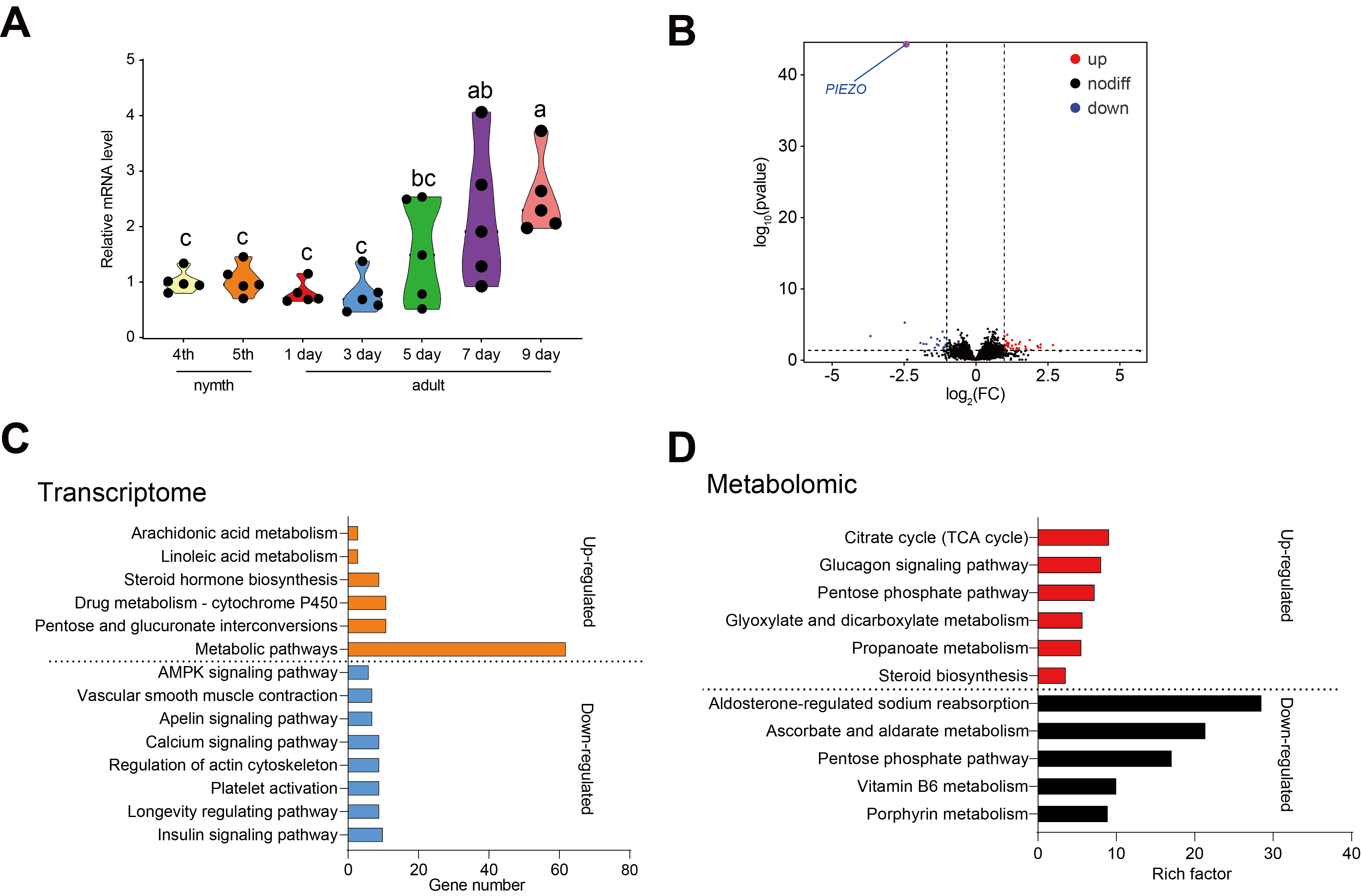


Figure. S25. Differential expression and influence mechanism analysis of the *PIEZO*. (A) Expression amount of *PIEZO* at different instars and different days after emergence. The values are mean ± SD. (n = 5 replicates, five locusts per replicate), one-way ANOVA, different lowercase letters above the bars indicate a significant difference (*p* < 0.05). (B) Volcano analysis of flight muscle. Transcriptome of flight muscle was sequenced using five biological replicates of five locusts. Only gene with *p* < 0.05 are shown. Red point represents gene upregulated expression, blue represents gene downregulated expression, and the black signal represents no significant difference. Expression levels were measured through transcriptome sequencing. (C) KEGG enrichment analysis of differentially expressed genes caused by *PIEZO* expression knockdown. Transcriptome of flight muscle was sequenced using five biological replicates of five locusts. Only KEGG terms with *p* < 0.05 are shown. (D) KEGG enrichment analysis of differentially expressed metabolites caused by *PIEZO* expression knockdown. Metabolome of flight muscle was measured using seven biological replicates of five locusts. Only KEGG terms with *p* < 0.05 are shown.

**Legends for Table**

**Table S1. Summary of genome assembly and annotation for *Locusta migratoria*.**

**Table S2. Statistics for chromosome-level scaffolding of the locust genome.**

**Table S3. Summary of genes with at least one exon having high repeat level in locust.**

**Table S4. Fold change in total TE copy percentage.**

**Table S5. Occurrence frequency of highest abundance TE families within six TE superfamilies.**

**Table S6. Locust sample information.**

**Table S7. Summary of whole-genome sequencing data.**

**Table S8. Summary of RNA sequencing data.**

**Table S9. Statistics of variation information on each chromosome.**

**Table S10. Candidate genes under selection identified by Z*fst*.**

**Table S11. Candidate genes under selection identified by XP-CLR.**

**Table S12. Candidate genes identified by the intersection of XP-CLR, Z*fst*, and nSL analyses.**

**Table S13. GO enrichment analysis of gene in the top 5% of nSL, Z*fst* and XP-CLR region.**

**Table S14. Total number of significant SNPs and putatively linked TEs for each haplotype-based analysis.**

**Table S15. Genes influenced by candidate adaptive TEs.**

**Table S16. Statistics of candidate adaptive TEs information on each chromosome.**

**Table S17. Classification and frequency of genomic TE and candidate adaptive TE.**

**Table S18. Chi-square test of genomic TE and candidate adaptive TE frequencies.**

**Table S19. Candidate genes under selective sweeps identified by the three methods.**

**Table S20. List of 279 genes co-selected across three approaches (XP-CLR, Z*fst*, and adaptive TE association).**

**Table S21. The PiggyBac insertion in different latitudes locust sample information.**

**Table S22. Primers used in this study.**
